# The role of geography and distance on physician follow-up after a first hospitalization with a diagnosis of a schizophrenia spectrum disorder: A retrospective population-based cohort study in Ontario, Canada

**DOI:** 10.1371/journal.pone.0287334

**Published:** 2023-06-16

**Authors:** Martin Rotenberg, Peter Gozdyra, Kelly K. Anderson, Paul Kurdyak

**Affiliations:** 1 Centre for Addiction and Mental Health, Toronto, Ontario, Canada; 2 Department of Psychiatry, University of Toronto, Toronto, Ontario, Canada; 3 ICES, Toronto, Ontario, Canada; 4 Department of Epidemiology and Biostatistics, Western University, London, Ontario, Canada; 5 Department of Psychiatry, Western University, London, Ontario, Canada; Università degli Studi di Milano, ITALY

## Abstract

**Background:**

Timely follow-up after hospitalization for a schizophrenia spectrum disorder (SSD) is an important quality indicator. We examined the proportion of individuals who received physician follow-up within 7 and 30 days post-discharge by health region and estimated the effect of distance between a person’s residence and discharging hospital on follow-up.

**Methods:**

We created a retrospective population-based cohort of incident hospitalizations with a discharge diagnosis of a SSD between 01/01/2012 and 30/03/2019. The proportion of follow-up with a psychiatrist and family physician within 7 and 30 days were calculated for each region. The effect of distance between a person’s residence and discharging hospital on follow-up was estimated using adjusted multilevel logistic regression models.

**Results:**

We identified 6,382 incident hospitalizations for a SSD. Only 14.2% and 49.2% of people received follow-up care with a psychiatrist within 7 and 30 days of discharge, respectively, and these proportions varied between regions. Although distance from hospital was not associated with follow-up within 7 days of discharge, increasing distance was associated with lower odds of follow-up with a psychiatrist within 30 days.

**Conclusion:**

Post-discharge follow-up is poor across the province. Geospatial factors may impact post-discharge care and should be considered in further evaluation of quality of care.

## Introduction

People in the early phase of a schizophrenia spectrum disorder (SSD) can benefit from timely interventions and follow-up to reduce the risk of poor outcomes, including hospitalization, poor treatment adherence, substance misuse and mortality [1, 2]. Those who are hospitalized at the time of first diagnosis may be at even greater risk of poor outcomes, as recent population-based data has described these people are over 13 times more likely to die within 5 years of diagnosis compared with age-matched, population-based controls due to suicide and unnatural deaths [3]. Quality standards in both the USA [4] and Canada [5] have focused on post-discharge quality of care to address the problem of lengthy times to post-discharge follow up after a SSD-related hospitalization. In Ontario (Canada) timely outpatient follow-up within 7-days of an inpatient hospitalization discharge for a SSD is a quality indicator [5]. Timely follow-up with a physician is important to monitor response and tolerability to treatment, assess safety, and support recovery as a person transitions back to the community following hospitalization.

At the present time we do not know what proportion of people who are discharged from hospital after a first diagnosis of SSD are seen within 7 days. Previous work from Ontario, Canada has estimated that only 45% of people were seen by a psychiatrist within 30 days of discharge [6]. There is limited data on how other factors, specifically geographic factors may impact timely follow-up and quality of care for people with a SSD.

A previous meta-analysis of health service use and distance has shown that geographical factors impact health service use, and people who live closer to healthcare facilities have better health related outcomes [7]. Although only three of the 108 studies included heterogenous samples of a variety of mental disorder including SSDs, the three studies included [8–10] all found a distance decay effect, whereby health service use outcomes in heterogenous samples with mental disorders were inversely associated with how far a person resides from services. Additional studies that have recently been published focusing on people with a SSD in Nigeria [11], Taiwan [12] and the USA [13] which have found distance to hospital impact the likelihood of missing a follow-up clinic appointment post-discharge [11], impacted length of stay [12], and be associated with rehospitalization [13]. None of the previous studies focused on first episode psychosis. There is a dearth of research examining how geospatial factors like distance to services may impact service use and quality of care during an early phase of a SSD, particularly during the potentially high-risk period following hospitalization discharge.

The objectives of this study were to 1) estimate the proportion of people discharged with SSD in Ontario who received timely-follow up while accounting for geographical factors and 2) examine the association between distance from a person’s place of residence to hospital and timely follow-up while adjusting for potential confounding factors.

For the purpose of this study, we examined distance from hospital to a person’s residence (as opposed to a local outpatient service or provider) as based on previous work more than half of people in previous population-based cohorts in the province were not seen by outpatient services after discharge [6]. Moreover, nearly all the specialized early psychosis intervention programs in the province are either hospital-based or located very close to a local hospital [14]. Hospitals would also be most familiar with relevant services that are within local health regions and have more established referral pathways to services that are closer.

## Methods

### Study design

We used administrative health data from ICES to construct a retrospective cohort of Ontario residents ages 16 to 40 years who were discharged from a hospitalization with an incident diagnosis of SSD between January 1, 2012 and March 31, 2019. People were followed up for 30 days after hospital discharge. These ages were used as they 1) overlap with many of the early psychosis intervention program age criteria in Ontario, and 2) provide coverage beyond age 30 which is closer to the actual the mean age of diagnosis of a SSD in Ontario (32.5 years) [15]. The follow-up period was prior to the onset of the COVID-19 pandemic during which there was a rapid update of virtual care [16].

### Setting

Ontario is a Canadian province with a population of approximately 14 million people and has a single-payer universal healthcare system. The province is approximately 1 million km^2^ and has a large geographic spread. Historically there have been 14 Local Health Integration Network (LHIN) regions (S1 Appendix) that range in size and contain 1 to 2 million people and have been used for the purposes of integration, funding, and planning health care. The LHINs are further subdivided into 97 primary sub-LHINs. For this study, we used LHIN regions and sub-LHIN regions for geospatial analyses and multi-level modeling.

### Data

The datasets used were linked using unique encoded identifiers and analyzed at ICES. ICES is an independent, non-profit research institute whose legal status under Ontario’s health information privacy law allows it to collect and analyze health care and demographic data, without consent, for health system evaluation and improvement.

The data sources included the Registered Persons Database; the Ontario Mental Health Reporting System (OMHRS); the Canadian Institute for Health Information Discharge Abstract Database; the National Ambulatory Care Reporting System; the Ontario Health Insurance Plan (OHIP) claims database; the ICES Physician Database; information about Ontario health care institutions funded by the Ministry of Health and Long-Term Care; Immigration, Refugees, and Citizenship Canada Permanent Residents database; the Ontario Marginalization Index; the Postal Code Conversion File; Ontario Road Network; and the Canadian Census of Population. Further details on the data sources are presented in S2 Appendix.

### Case definition

We ascertained incident or “first-episode” cases of SSD during a hospitalization by ensuring that people included in the cohort had no previous SSD diagnosis. We included all first hospitalizations with a discharge diagnosis of Schizophrenia, Schizoaffective Disorder and Psychotic Disorder NOS based on DSM-IV / ICD-9 codes (295,298) and ICD-10-CA codes (F20, F25, F29) [17]. All people included in the cohort were residents of Ontario and eligible for OHIP coverage, had at least a 5-year lookback period to ascertain if cases were incident, had a hospitalization length of stay greater than 3 days to ensure sufficient time for diagnosis, and had a length of stay no longer than 90 days as long stays are not common in this clinical population. We also excluded forensic hospitalizations as outpatient care pathways differ in the forensic system. Finally, we restricted our regression models to a subset of the cohort who were hospitalized in designated mental health beds that reported detailed clinical assessment to OMHRS, because this contains information on clinical factors important for understanding physician follow-up [11, 18–22].

### Exposure: Distance

The exposure of interest was the straight line (Euclidian) distance between the discharging hospital and a person’s place of residence. This was calculated based on location of residence at the time of hospitalization discharge and the location of the hospital.

### Outcomes: Physician follow-up

Our primary outcome was defined as follow-up vs. no follow-up with a psychiatrist within 7 days post-discharge. We focused on the 7-day time window to reflect Ontario’s quality standard for follow-up for people with SSD post-discharge.

Our secondary outcomes included: follow-up vs. no follow-up with a psychiatrist within 30 days post-discharge, follow-up vs. no follow-up with any physician (psychiatrist or FP) within 7 days post-discharge, and follow-up vs. no follow-up with any physician within 30 days post-discharge.

FP follow-up was included as part of our secondary outcomes as FPs play an important role in providing mental health services to this population in Ontario [23]. Considering FPs may provide mental health services as part of a general medical visit [24] specific mental health billing codes were not used, and any visit to a FP during the follow-up period was included.

### Covariates

We included covariates that were known to impact physician follow-up in this population [6, 11, 18–22, 25] and were available in the health administrative data. Further details on the rational for covariate inclusion, covariate categorization and the conceptual framework are included in S3 Appendix.

Sociodemographic factors included age, sex, immigrant status, housing stability, and living arrangements.

Socioenvironmental factors included rural residence based on the Rurality Index of Ontario [26] and area-level marginalization based on the Ontario Marginalization Index (ON-Marg) which includes material deprivation, residential instability, dependency and ethnic concentration dimensions [27]. We also assigned all people to one of the 14 LHINs and 97 primary sub-LHINs based on residence at the time of discharge.

Clinical factors included diagnosis (dichotomized as either schizophrenia/schizoaffective disorder or psychosis NOS), length of stay in hospital, as well as history of problematic substance use, Positive Symptom Scale (PSS), involuntary admission, and insight (full, limited or no insight) which were based on RAI-MH assessment data.

At the hospital level we differentiated between community and academic hospitals. From a service use perspective, we identified any previous contact in the year prior to hospitalization with a psychiatrist, with a FP, any previous ED visits for any mental health reason, and any mental health hospitalization for a reason other than a SSD. Temporally, we also captured the year of discharge to account for potential changes in the structure and funding of mental health services during the study period.

### Analysis

We first calculated the crude proportion of people in the entire cohort and in each LHIN for each outcome. Considering that crude proportions do not account for differences in population size across the LHINs, we used the 2016 population estimate in each LHIN based on the 2016 Canadian Census to calculate standardized event ratios (SER). We visualized SER for each outcome of interest using choropleth maps at the LHIN-level for the entire province. A SER less than 1 indicates there was a lower proportion of people with physician follow-up in a region than expected, and a SER greater than 1 indicates there was a larger proportion of people with follow-up than expected.

We described the characteristics of the analytic cohort stratified by our primary outcome using standardized differences [28].

We used a log base 2 transformation of the distance variable due to the wide range and right-skewed distribution. The odds ratio (OR) of this transformation can be interpreted as the change in the odds for every 2-fold increase in distance. Considering the transformed variable was linearly associated with the logit of the outcome, did not change the statistical significance of the exposure estimates, and did not change the estimates of other variables in the models, we reported the log base 2 transformation for all models.

We first fit multilevel logistic regression models for all outcomes of interest with random intercepts at the sub-LHIN region level and no predictor variables. Random intercepts were fit at the sub-LHIN region level to account for the nested structure (people receiving care within health administrative regions) and clustering within these smaller geographic regions. All further multilevel models were fit with random intercepts at the sub-LHIN level.

We used the null models with only the random intercept and no predictor variables to estimate general contextual effects, which provide information on between-cluster variances at the sub-LHIN region level for all outcomes of interest. We calculated intraclass correlation coefficients (ICC) to quantify the variation in the outcomes attributable to the sub-LHIN level [29]. Median odds ratio (MOR) were also calculated and can be interpreted as the median increase in the odds ratio as one moves from an area with a low proportion of follow-up to a high proportion area [30, 31].

We then proceeded to fit unadjusted multilevel logistic regression models and then fit adjusted models for all outcomes of interest with our exposure of interest and covariates. The null models and unadjusted models fit with the primary exposure were used to assess the fit of the fully adjusted models. All effect estimates were presented as ORs with 95% confidence intervals. Estimates were considered significant at the p<0.05 level when the confidence intervals did not overlap with unity.

We conducted sensitivity analyses to assess the impact of potential exposure misclassification, which included comparing model estimates with alternative ways of measuring the primary exposure of interest, including drive time and driving distance from hospital to a person’s residence. Additional sensitivity analyses restricted the sample based on housing stability and extreme distances.

We used R 3.1.2 [32] to conduct descriptive analyses and to visualize data, Stata 15.1 [33] for regression analyses, and ArcGIS 10.2 [34] for network analyses for the sensitivity analyses.

### Ethics approval

The study was approved by the Centre for Addiction and Mental Health Research Ethics Board, Toronto, Canada. Individual informed consent was waived. The study data was de-identified prior to analysis.

## Results

We identified 25,637 total hospitalizations for SSD between January 1, 2012 and March 31, 2019. Of these, 6,382 hospitalizations were for an incident diagnosis of SSD and met the inclusion criteria and were included in the descriptive analysis. Of these hospitalizations 5,882 were discharges from a psychiatric inpatient unit and were included in the analytic sample that used detailed clinical data. The characteristics of the full cohort are presented in Table 1, and the characteristics of the analytic sample stratified by the primary outcome are presented in S4 Appendix.

**Table 1:** Characteristics of all people with an incident diagnosis of a schizophrenia spectrum disorder (SSD) diagnosed during a first hospitalization in Ontario between Jan 1, 2012 and March 30, 2019 (n = 6,382).

Variable	n (%)*
Distance (median [IQR]) ^a^	9.5 [4.0, 23.5]
Age (median [IQR])	24.0 [20.0, 30.0]
Male	4246 (66.5)
Immigration History	
General Population	5340 (83.7)
Immigrant	772 (12.1)
Refugee	270 (4.2)
Psychosis NOS	3808 (59.7)
Length of stay (median [IQR])	14.00 [9.00, 23.00]
Teaching hospital	1931 (30.3)
Rural residence ^b^	676 (10.7)
Area-level marginalization	
Dependency ^c^	
Q1 (low)	1687 (27.2)
Q2	1340 (21.6)
Q3	1115 (18.0)
Q4	1014 (16.3)
Q5 (high)	1054 (17.0)
Deprivation ^c^	
Q1 (low)	942 (15.2)
Q2	956 (15.4)
Q3	1140 (18.4)
Q4	1313 (21.1)
Q5 (high)	1859 (29.9)
Ethnic concentration ^c^	
Q1 (low)	952 (15.3)
Q2	1016 (16.4)
Q3	1041 (16.8)
Q4	1236 (19.9)
Q5 (high)	1965 (31.6)
Residential instability ^c^	
Q1 (low)	1035 (16.7)
Q2	1025 (16.5)
Q3	1036 (16.7)
Q4	1322 (21.3)
Q5 (high)	1792 (28.9)
Psychiatrist contact (in prev. year)	1567 (24.6)
Mental health contact with FP (in prev. year)	2827 (44.3)
Mental health ED visit (in prev. year)	2838 (44.5)
Mental health hospitalization (in prev. year)	588 (9.2)

otes: * n (%) unless otherwise specified

^a^ 50 (0.7%) missing observations

^b^ 52 (0.8%) missing observations

^c^ 173 (2.7%) missing observations

Only 14% of the analytic cohort received psychiatric follow-up within 7 days post-discharge. The median distance between a person’s residence and the discharging hospital was 8.0 km (IQR: 3.8 km, 18.4 km) in the group that received follow-up with a psychiatrist within 7 days post-discharge, compared to 9.71 km (IQR: 3.9 km, 24.09 km) in the group that did not receive follow-up.

A greater median length of stay was observed for people who had follow-up with a psychiatrist post-discharge. We observed a greater proportion of people who were admitted to hospital involuntarily in the group that received follow-up by a psychiatrist. Furthermore, a greater proportion of people in the group who were seen by a psychiatrist within 7 days resided in urban areas with greater levels of ethnic concentration. We also observed a greater proportion of people admitted to academic hospitals who were seen by a psychiatrist within 7 days post-discharge. With respect to previous service use, a greater proportion of people who received follow-up with a psychiatrist had previous contact with a psychiatrist and FPs within 1 year prior to hospitalization.

### Follow-up by Local Health Integration Network

Only 14.2% of the entire cohort had a visit with a psychiatrist within 7 days post-discharge. The crude proportion of the cohort receiving follow-up differed between LHINs and ranged between 4.8% to 27.6% of the cohort receiving follow-up with a psychiatrist within 7 days post-discharge. Almost half of the entire cohort (49.6%) were seen by a psychiatrist within 30 days post-discharge, and this ranged between 17.7% to 63.2% based on region.

**Fig 1:**
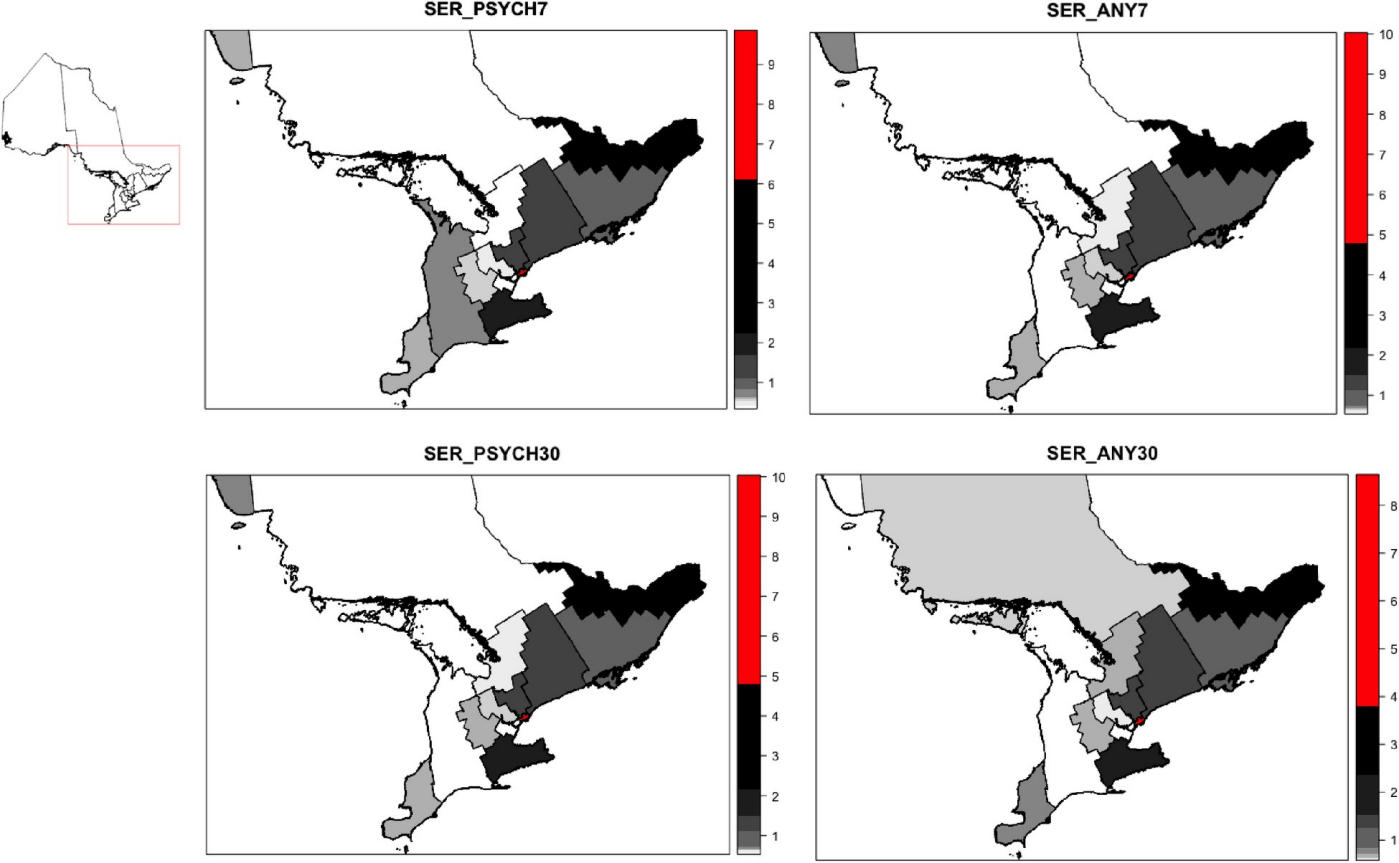
Maps of standardized event ratios of follow-up with a psychiatrist or any physician within 7 and 30 days of hospitalization discharge by Local Health Integration Network region. Notes: LHINs with the top 10% SERs are highlighted in red, SER = standardized event ratios, PSYCH7 = Follow-up with a psychiatrist within 7 days of hospitalization discharge, PYCH30 = Follow-up with a psychiatrist within 30 days of hospitalization discharge, ANY7 = Follow-up with any physician within 7 days of hospitalization discharge, ANY30 = Follow-up with any physician within 30 days of hospitalization discharge. *This figure contains information licensed under the Open Government Licence–Ontario, https://www.ontario.ca/page/open-government-licence-ontario.

Visualization of the SERs for all outcomes by LHIN are presented in Fig 1. Overall, there was a similar pattern across all outcomes, with a greater than expected level of follow-up in the LHINs with the largest urban centers (Toronto Central LHIN, followed by the Champlain and the Hamilton-Niagara-Haldimand-Brant LHINs).

### Contextual effects at the regional level

An ICC of 0.10 (95% CI = 0.05 to 0.16) indicated that 10% of the variance occurred at the sub-LHIN region level for the primary outcome, follow-up with a psychiatrist within 7 days. A MOR of 1.80 indicated that the sub-LHIN regions with the greatest odds of follow-up had an 80% increased odds of follow-up when compared to the regions with the lowest odds of follow-up. ICCs for secondary outcomes ranged between 0.15 (95% CI = 0.11 to 0.21) for follow-up with a psychiatrist within 30 days and 0.03 (95% CI = 0.01 to 0.06) for FP follow-up within 7 days. MORs ranged between 2.07 for follow-up with a psychiatrist within 30 days and 1.33 for FP follow-up within 7 days.

### The association between distance and follow-up

The unadjusted and fully adjusted estimates of the primary effect of distance from residence to hospital on the primary and secondary outcomes are presented in Table 2. Full model estimates, including secondary effects, are presented in Tables 3–6.

**Table 2:** Unadjusted and adjusted odds ratios of log base 2 transformation of distance from all models (n = 5,590).

Model	Unadjusted OR	95% CI	Adjusted OR	95% CI
Psychiatrist follow-up within 7 days	0.99	0.93–1.02	1.00	0.95–1.05
Psychiatrist follow-up within 30 days	**0.95**	**0.92–0.98**	**0.96**	**0.92–0.99**
Any physician follow-up within 7 days	0.99	0.95–1.02	1.01	0.97–1.05
Any physician follow-up within 30 days	0.97	0.94–1.01	0.98	0.95–1.02

Note: Significant results are bolded

**Table 3:** Unadjusted and adjusted mixed logistic regression models of factors associated with psychiatrist follow-up within 7 days of hospitalization discharge (n = 5,590).

Variable	Value	Unadjusted OR	95% CI	Adjusted OR	95% CI
Distance (Log base 2)	2x increase in distance	0.99	0.93–1.02	1.00	0.95–1.05
Age	(years)	**0.99**	**0.97–0.99**	0.99	0.97–1.00
Sex	Female	Reference		Reference	
	Male	**0.84**	**0.72–0.99**	0.86	0.72–1.02
Migrant status	General population	Reference		Reference	
	Immigrant	0.85	0.67–1.07	0.87	0.68–1.12
	Refugee	0.83	0.57–1.22	0.87	0.58–1.30
Housing stability	Stable	Reference		Reference	
	Not stable	1.06	0.86–1.23	0.96	0.80–1.16
Lives alone	No	Reference		Reference	
	Yes	0.89	0.68–1.01	0.84	0.68–1.05
Residence	Urban	Reference		Reference	
	Rural	**0.59**	**0.41–0.84**	0.72	0.48–1.10
Dependency	Q1 (low)	Reference		Reference	
	Q2	1.06	0.85–1.33	1.13	0.89–1.41
	Q3	1.20	0.94–1.52	**1.31**	**1.02–1.68**
	Q4	1.08	0.84–1.40	1.22	0.93–1.61
	Q5 (high)	0.98	0.76–1.28	1.18	0.89–1.57
Deprivation	Q1 (low)	Reference		Reference	
	Q2	0.95	0.73–1.25	0.98	0.74–1.29
	Q3	0.92	0.70–1.20	0.92	0.70–1.22
	Q4	0.84	0.64–1.10	0.85	0.63–1.13
	Q5 (high)	0.85	0.66–1.09	0.84	0.62–1.14
Ethnic concentration	Q1 (low)	Reference		Reference	
	Q2	0.97	0.79–1.51	0.98	0.70–1.37
	Q3	**1.58**	**1.15–2.16**	1.36	0.97–1.90
	Q4	**1.76**	**1.28–2.41**	**1.59**	**1.12–2.25**
	Q5 (high)	**1.45**	**1.05–2.01**	**1.48**	**1.02–2.15**
Residential instability	Q1 (low)	Reference		Reference	
	Q2	0.89	0.66–1.18	0.85	0.63–1.14
	Q3	1.06	0.80–1.40	1.06	0.79–1.42
	Q4	0.92	0.71–1.22	1.00	0.74–1.35
	Q5 (high)	0.92	0.71–1.20	0.97	0.72–1.31
Diagnosis	Schizophrenia	Reference		Reference	
	Psychosis NOS	0.97	0.83–1.13	1.06	0.90–1.25
PSS	(0–24)	0.99	0.97–1.02	1.01	0.99–1.04
Admission	Involuntary	Reference		Reference	
	Voluntary	**1.68**	**1.36–2.07**	**1.40**	**1.12–1.75**
Substance use	No	Reference		Reference	
	Yes	**0.79**	**0.68–0.92**	**0.84**	**0.72–0.99**
Insight	Full	Reference		Reference	
	Limited	0.85	0.68–1.06	0.91	0.73–1.14
	None	0.80	0.63–1.03	0.89	0.68–1.16
LOS	(days)	**1.01**	**1.01–1.02**	**1.01**	**1.00–1.02**
Teaching hospital	No	Reference		Reference	
	Yes	**1.91**	**1.57–2.32**	**1.58**	**1.29–1.93**
Mental health hospitalization (in prev. year)	No	Reference		Reference	
	Yes	1.09	0.83–1.42	0.84	0.62–1.13
Mental health ED visit (in prev. year	No	Reference		Reference	
	Yes	1.00	0.86–1.17	0.83	0.63–1.09
Psychiatrist contact (in prev. year)	No	Reference		Reference	
	Yes	**1.94**	**1.65–2.29**	**1.56**	**1.24–1.97**
Mental health contact with FP (in prev. year)	No	Reference		Reference	
	Yes	**1.26**	**1.09–1.47**	1.09	0.89–1.32
Year	2012–2019	0.95	0.92–0.99	**0.94**	**0.91–0.98**

Notes: Significant results are bolded, Q = quintile, PSS = Positive Symptom Scale, LOS = length of stay, FP = family physician

**Table 4:** Unadjusted and adjusted mixed logistic regression models of factors associated with psychiatrist follow-up within 30 days of hospitalization discharge (n = 5,590).

Variable	Value	Unadjusted OR	95% CI	Adjusted OR	95% CI
Distance (Log base 2)	2x increase in distance	**0.95**	**0.92–0.98**	**0.96**	**0.92–0.99**
Age	(years)	**0.98**	**0.97–0.99**	**0.98**	**0.97–0.99**
Sex	Female	Reference		Reference	
	Male	0.96	0.86–1.10	0.97	0.86–1.10
Migrant status	General population	Reference		Reference	
	Immigrant	0.98	0.83–1.16	1.02	0.85–1.22
	Refugee	0.85	0.64–1.10	0.88	0.66–1.16
Residential stability	Stable	Reference		Reference	
	Not stable	1.01	0.89–1.14	1.00	0.87–1.15
Lives alone	No	Reference		Reference	
	Yes	**0.66**	**0.57–0.76**	**0.70**	**0.60–0.81**
Residence	Urban	Reference		Reference	
	Rural	**0.62**	**0.49–0.79**	**0.73**	**0.55–0.96**
Dependency	Q1 (low)	Reference		Reference	
	Q2	0.95	0.81–1.12	0.95	0.81–1.13
	Q3	1.07	0.90–1.26	1.07	0.89–1.30
	Q4	0.93	0.78–1.12	0.93	0.76–1.13
	Q5 (high)	0.96	0.80–1.16	1.06	0.86–1.31
Deprivation	Q1 (low)	Reference		Reference	
	Q2	0.91	0.74–1.11	0.92	0.74–1.13
	Q3	0.91	0.75–1.11	0.92	0.75–1.14
	Q4	0.84	0.69–1.02	0.90	0.72–1.11
	Q5 (high)	**0.67**	**0.55–0.81**	**0.70**	**0.56–0.87**
Ethnic concentration	Q1 (low)	Reference		Reference	
	Q2	1.19	0.97–1.45	1.11	0.89–1.38
	Q3	1.32	1.06–1.64	1.20	0.95–1.52
	Q4	**1.37**	**1.10–1.71**	**1.29**	**1.01–1.65**
	Q5 (high)	**1.28**	**1.01–1.62**	**1.36**	**1.03–1.79**
Residential instability	Q1 (low)	Reference		Reference	
	Q2	1.17	0.96–1.43	1.20	0.98–1.48
	Q3	1.06	0.87–1.30	1.10	0.88–1.35
	Q4	0.92	0.76–1.12	1.03	0.84–1.28
	Q5 (high)	0.85	0.71–1.-3	0.98	0.79–1.22
Diagnosis	Schizophrenia	Reference		Reference	
	Psychosis NOS	0.87	0.78–0.98	0.98	0.87–1.10
PSS	(0–24)	0.99	0.97–1.00	0.99	0.98–1.02
Admission	Involuntary	Reference		Reference	
	Voluntary	**1.35**	**1.14–1.60**	1.18	0.99–1.42
Substance use	No	Reference		Reference	
	**Yes**	**0.79**	**0.71–0.88**	**0.84**	**0.74–0.94**
Insight	Full	Reference		Reference	
	Limited	0.87	0.74–1.02	0.87	0.73–1.03
	None	**0.79**	**0.66–0.94**	**0.78**	**0.64–0.95**
LOS	(days)	**1.02**	**1.01–1.02**	**1.02**	**1.01–1.02**
Teaching hospital	No	Reference		Reference	
	Yes	**1.35**	**1.16–1.58**	1.11	0.94–1.31
Mental health hospitalization (in prev. year)	No	Reference		Reference	
	Yes	0.86	0.71–1.05	**0.64**	**0.51–0.80**
Mental health ED visit (in prev. year	No	Reference		Reference	
	Yes	1.03	0.92–1.14	0.90	0.73–1.09
Psychiatrist contact (in prev. year)	No	Reference		Reference	
	Yes	**1.81**	**1.60–2.06**	**1.40**	**1.17–1.67**
Mental health contact with FP (in prev. year	No	Reference		Reference	
	Yes	**1.24**	**1.12–1.49**	1.05	0.91–1.21
Year	2012–2019	**0.96**	**0.94–0.99**	**0.96**	**0.93–0.99**

Notes: Significant results are bolded, Q = quintile, PSS = Positive Symptom Scale, LOS = length of stay, FP = family physician

**Table 5:** Unadjusted and adjusted mixed logistic regression models of factors associated with any physician follow-up within 7 days of hospitalization discharge (n = 5,590).

Variable	Value	Unadjusted OR	95% CI	Adjusted OR	95% CI
Distance (Log base 2)	2x increase in distance	0.99	0.95–1.02	1.01	0.97–1.05
Age	(years)	1.01	0.99–1.02	1.01	0.99–1.02
Sex	Female	Reference		Reference	
	Male	**0.86**	**0.76–0.97**	0.93	0.81–1.06
Migrant status	General population	Reference		Reference	
	Immigrant	0.99	0.82–1.20	1.03	0.84–1.25
	Refugee	0.81	0.60–1.11	0.81	0.59–1.13
Residential stability	Stable	Reference		Reference	
	Not stable	0.92	0.80–1.10	0.90	0.77–1.04
Lives alone	No	Reference		Reference	
	Yes	0.86	0.74–1.01	**0.84**	**0.71–0.99**
Residence	Urban	Reference		Reference	
	Rural	**0.65**	**0.51–0.84**	0.78	0.58–1.04
Dependency	Q1 (low)	Reference		Reference	
	Q2	0.98	0.82–1.17	1.02	0.84–1.22
	Q3	1.12	0.93–1.35	1.17	0.95–1.42
	Q4	0.93	0.76–1.14	0.98	0.79–1.23
	Q5 (high)	0.99	0.81–1.22	1.09	0.87–1.36
Deprivation	Q1 (low)	Reference		Reference	
	Q2	0.99	0.80–1.24	1.00	0.79–1.26
	Q3	1.02	0.82–1.26	1.04	0.83–1.31
	Q4	0.92	0.75–1.14	0.97	0.77–1.22
	Q5 (high)	0.85	0.68–1.04	0.91	0.72–1.16
Ethnic concentration	Q1 (low)	Reference		Reference	
	Q2	1.24	0.98–1.57	1.17	0.92–1.51
	Q3	1.52	1.20–1.92	**1.42**	**1.09–1.84**
	Q4	**1.53**	**1.21–1.95**	**1.50**	**1.14–1.96**
	Q5 (high)	1.26	0.99–1.62	**1.34**	**1.00–1.79**
Residential instability	Q1 (low)	Reference		Reference	
	Q2	1.02	0.82–1.28	0.99	0.79–1.25
	Q3	**1.25**	**1.00–1.56**	**1.26**	**1.00–1.59**
	Q4	1.02	0.82–1.26	1.08	0.86–1.38
	Q5 (high)	0.97	0.79–1.20	1.02	0.81–1.29
Diagnosis	Schizophrenia	Reference		Reference	
	Psychosis NOS	1.12	0.98–1.25	1.12	0.98–1.28
PSS	(0–24)	0.99	0.98–1.02	1.01	0.99–1.03
Admission	Involuntary	Reference		Reference	
	Voluntary	**1.41**	**1.18–1.68**	1.18	0.98–1.42
Substance use	No	Reference		Reference	
	Yes	0.92	0.81–1.04	0.99	0.87–1.13
Insight	Full	Reference		Reference	
	Limited	0.87	0.73–1.04	0.90	0.75–1.08
	None	**0.74**	**0.61–0.91**	0.82	0.66–1.01
LOS	(days)	**1.01**	**1.00–1.01**	**1.01**	**1.00–1.01**
Teaching hospital	No	Reference		Reference	
	Yes	**1.44**	**1.23–1.68**	**1.28**	**1.09–1.51**
Mental health hospitalization (in prev. year)	No	Reference		Reference	
	Yes	1.06	0.86–1.31	0.94	0.74–1.19
Mental health ED visit (in prev. year	No	Reference		Reference	
	Yes	1.08	0.96–1.23	0.80	0.64–1.00
Psychiatrist contact (in prev. year)	No	Reference		Reference	
	Yes	**1.45**	**1.26–1.66**	1.17	0.96–1.42
Mental health contact with FP (in prev. year)	No	Reference		Reference	
	Yes	**1.75**	**1.55–1.97**	**1.30**	**1.11–1.52**
Year	2012–2019	**0.98**	**0.96–1.01**	**0.97**	**0.94–0.99**

Notes: Significant results are bolded, Q = quintile, PSS = Positive Symptom Scale, LOS = length of stay, FP = family physician

**Table 6:** Unadjusted and adjusted mixed logistic regression models of factors associated with any physician follow-up within 30 days of hospitalization discharge (n = 5,590).

Variable	Value	Unadjusted OR	95% CI	Adjusted OR	95% CI
Distance (Log base 2)	2x increase in distance	0.97	0.94–1.01	0.98	0.95–1.02
Age	(years)	1.01	0.99–1.02	1.01	0.99–1.02
Sex	Female	Reference		Reference	
	Male	**0.81**	**0.72–0.91**	0.89	0.78–1.01
Migrant status	General population	Reference		Reference	
	Immigrant	1.11	0.93–1.33	1.12	0.92–1.36
	Refugee	0.79	0.60–1.04	0.79	0.59–1.06
Residential stability	Stable	Reference		Reference	
	Not stable	0.94	0.82–1.07	0.96	0.83–1.10
Lives alone	No	Reference		Reference	
	Yes	**0.76**	**0.66–0.88**	**0.77**	**0.66–0.90**
Residence	Urban	Reference		Reference	
	Rural	**0.66**	**0.53–0.82**	0.81	0.63–1.05
Dependency	Q1 (low)	Reference		Reference	
	Q2	0.87	0.73–1.02	0.86	0.72–1.03
	Q3	0.94	0.79–1.12	0.94	0.77–1.14
	Q4	**0.83**	**0.68–0.99**	0.82	0.67–1.01
	Q5 (high)	0.88	0.72–1.06	0.96	0.77–1.19
Deprivation	Q1 (low)	Reference		Reference	
	Q2	0.90	0.72–1.11	0.90	0.72–1.13
	Q3	0.98	0.79–1.21	1.03	0.82–1.28
	Q4	**0.75**	**0.61–0.93**	0.83	0.66–1.04
	Q5 (high)	**0.65**	**0.53–0.79**	**0.72**	**0.57–0.90**
Ethnic concentration	Q1 (low)	Reference		Reference	
	Q2	**1.30**	**1.06–1.59**	1.22	0.98–1.51
	Q3	**1.34**	**1.08–1.66**	1.23	0.98–1.56
	Q4	**1.37**	**1.10–1.71**	**1.29**	**1.01–1.65**
	Q5 (high)	**1.29**	**1.02–1.62**	**1.35**	**1.03–1.77**
Residential instability	Q1 (low)	Reference		Reference	
	Q2	1.15	0.94–1.42	1.17	0.94–1.46
	Q3	1.13	0.92–1.40	1.18	0.94–1.47
	Q4	0.93	0.76–1.13	1.07	0.86–1.33
	Q5 (high)	0.83	0.69–1.01	0.94	0.75–1.18
Diagnosis	Schizophrenia	Reference		Reference	
	Psychosis NOS	1.01	0.90–1.13	1.05	0.93–1.20
PSS	(0–24)	0.99	0.97–1.01	1.01	0.99–1.03
Admission	Involuntary	Reference		Reference	
	Voluntary	**1.56**	**1.30–1.88**	**1.26**	**1.04–1.54**
Substance use	No	Reference		Reference	
	Yes	**0.76**	**0.68–0.85**	**0.85**	**0.75–0.96**
Insight	Full	Reference		Reference	
	Limited	**0.77**	**0.65–0.92**	**0.78**	**0.65–0.94**
	None	**0.64**	**0.53–0.79**	**0.68**	**0.55–0.84**
LOS	(days)	**1.01**	**1.01–1.02**	**1.01**	**1.01–1.02**
Teaching hospital	No	Reference		Reference	
	Yes	**1.40**	**1.19–1.64**	1.18	0.99–1.39
Mental health hospitalization (in prev. year)	No	Reference		Reference	
	Yes	0.89	0.73–1.08	**0.69**	**0.55–0.87**
Mental health ED visit (in prev. year)	No	Reference		Reference	
	Yes	1.08	0.97–1.21	0.84	0.68–1.03
Psychiatrist contact (in prev. year)	No	Reference		Reference	
	Yes	**1.68**	**1.47–1.94**	**1.22**	**1.01–1.48**
Mental health contact with FP (in prev. year)	No	Reference		Reference	
	Yes	**2.04**	**1.81–2.9**	**1.46**	**1.26–1.69**
Year	2012–2019	0.99	0.97–1.02	0.99	0.96–1.02

Notes: Significant results are bolded, Q = quintile, PSS = Positive Symptom Scale, LOS = length of stay, FP = family physician

### Primary effects

Distance was not associated with the primary outcome of follow-up with a psychiatrist within 7 days post-discharge in either the unadjusted (OR = 0.99, 95% CI = 0.93 to 1.02) or adjusted models (aOR = 1.00, 95% CI = 0.95 to 1.05). However, distance was independently associated with the secondary outcome of follow-up with a psychiatrist within 30 days post-discharge. In the fully adjusted model, there was a 4% reduction in the odds of follow-up for every 2-fold increase in distance between a person’s residence and location of hospitalization (aOR 0.96, 95% CI = 0.92 to 0.99). Distance was not associated with follow-up with any physician within 7 days post-discharge (aOR = 1.01, 95% CI = 0.97 to 1.05) or 30 days post-discharge (aOR = 0.98, 95% CI = 0.95 to 1.02).

### Secondary effects

The primary effect estimates (between distance and follow-up) were obtained from models constructed to obtain unbiased effect estimates of this relationship. Unadjusted and adjusted estimates for all covariates are also presented for completeness (Tables 3–6). These secondary effects must be interpreted with caution, as these estimates may be biased due to the models not being constructed to obtain unconfounded estimates of the relationship between the secondary exposures and outcomes for people with a history of substance use [35, 36].

In the adjusted model estimating the odds of psychiatric follow-up within 7 days of discharge (Table 3) there was a statistically significant increase in the odds of follow-up in areas with moderate levels of dependency (Q3), areas with higher levels of ethnic concentration (Q3,Q4), for people who were admitted voluntarily, admitted to a teaching hospital, had a longer length of stay and had previous psychiatric contact. There was a statistically significant reduction in the odds of follow-up with a psychiatrist within 7 days of discharge if a person had a history of substance use and with each increasing year in the cohort during which an admission occurred.

In the adjusted model for psychiatric follow-up within 3 days of discharge (Table 4) there was a statistically significant increase in the odds of follow-up in areas with higher levels of ethnic concentration (Q3,Q4), for people with a greater length of stay, and had previous contact with a psychiatrist prior to the hospitalization. As reported in the primary effects section, there was statistically significant and independent association between distance and follow-up with a psychiatrist within 30 days of follow-up, whereby there was a reduction in the in the odds of follow-up for every 2-fold increase in distance between a person’s residence and location of hospitalization. Furthermore, in the adjusted model there was also a statistically significant reduction in the odds of follow-up if a person lived alone, lived in a rural area, used substances, had no insight and had a previous mental health hospitalization not for a SSD, and with each increasing calendar year during the cohort period.

In the adjusted model estimating the odds of any physician follow-up within 7 days of discharge (Table 5) there was a statistically significant increase in the odds of follow-up in areas with moderate to high levels of ethnic concentration (Q3,Q4,Q5), moderate residential instability (Q3), in people with a longer length of stay in hospital, admitted to a teaching hospital and had previous contact with a family physician in the year prior to hospitalization. There was a statistically significant reduction in the odds of follow-up if a person lived alone and with each increasing calendar year during the cohort period.

In the adjusted model estimating any physician follow-up within 30 days of discharge there was a statistically significant increase in the odds of follow-up in areas with higher levels of ethnic concentration (Q4,Q5), and if a person was admitted as a voluntary patient, spent a longer time in hospital, and had contact with a psychiatrist or family physician for mental health reasons in the year prior to admission. There was a statistically significant reduction in the odds of follow-up if a person lived alone, in areas with higher levels of deprivation (Q5), if a person uses substances, has limited or no insight, and had a mental health hospitalization for a disorder other than a SSD in the year prior to the admission.

### Sensitivity analyses

We found no substantive change in estimates or significance when we compared models using Euclidian distance to models with alternative definitions of the exposure specifically, driving distance and driving time. Moreover, model estimates from sensitivity analyses that restricted the sample based on housing stability and extreme distances did not suggest misclassification of the primary exposure. Further details on the sensitivity analyses are presented in S5 Appendix.

## Discussion

Less than 3 out of every 20 people who are discharged from hospital in Ontario after a new diagnosis of SSD received follow-up with a psychiatrist within 7 days. Outpatient follow-up within 7 days post-discharge is the quality standard in Ontario for all people with schizophrenia during any phase of the illness [5]. Within 30 days post-discharge, slightly less than half of all people in the cohort were seen by a psychiatrist. In a similar clinical sample from the same jurisdiction but from an earlier time period (1999 and 2009) 44.8% of all people with a new diagnosis of SSD received outpatient follow-up with a psychiatrist within 30 days post-discharge [6]. Although our current study found a slight improvement in the proportion of people who received follow-up with a psychiatrist within 30 days post-discharge, it remains that less than half of all people who are hospitalized at first diagnosis with SSD receive follow-up care with a psychiatrist within 30 days post-discharge, and this trend has persisted over two decades.

Our findings also highlight disparities in post-discharge quality of care based on where a person resides in the province. The greatest proportion of people who received timely follow-up across all outcomes live in the health regions which include the largest metropolitan centers in the province (i.e., Toronto, Ottawa, and Hamilton).

The low proportion of follow-up we observed outside of the large metropolitan centers is in keeping with the unequal distribution of psychiatrists across the province [37]; however, other factors including the structure and delivery of mental health services must also be considered. From a policy perspective further consideration of the incentivization of quality care would be reasonable to examine despite initial negative studies [38]. Unlike previous work which has focused on broader populations, it would be warranted to study incentives targeted to specific clinical population based on service need and geography. It is unlikely that incentivization of care alone will improve quality, and alternative models to standard mental health care delivery that are both more efficient and responsive to the care needs of service users should also be considered.

We found no association between the distance from a person’s place of residence and the discharging hospital and the primary outcome of follow-up with a psychiatrist within 7 days post-discharge. However, we found a modest association between our secondary outcome of psychiatric follow-up within 30 days post-discharge with distance. These findings are suggestive of a distance decay effect when examined for specific types of physician follow-up within 30 days of hospitalization discharge. This finding is consistent with the literature on this topic from other areas of health care [7] and in similar clinical populations [8–10]. The fact that we did not find a significant association between distance and the outcomes that assessed care contacts within 7 days of discharge may be due to only a small proportion of the sample receiving follow-up within 7 days of hospitalization discharge. Moreover, it is possible that care received within 7 days of hospitalization discharge may be driven by factors other than distance, specifically factors like past service use, which may be more strongly associated with follow-up.

Further replication and examination is warranted to determine the degree of clinical significance of this distance decay finding, considering other factors (e.g., past service use and clinical factors) may be more strongly associated with follow-up. It may also be important to consider how distance may impact length of stay in hospital and the risk of rehospitalization, as previously examined in non-first episode cohorts of people with SSD diagnoses [12, 13]. Clinically, our results support the fact that greater care should be taken when discharging people from a first inpatient hospitalization who live further away from hospital and in specific regions where there may be poor access to services.

Several secondary exposures (ranging from area level marginalization, rurality, past psychiatric follow-up, family physician follow-up, substance use, level of insight, length of stay and year of admission) were associated with the odds of follow-up, it is important to note the adjusted models were built to obtain unbiased estimates of the primary exposure of distance on the outcomes of interest. These findings are not surprising and are in keeping with previous literature which has found sociodemographic, socioenvironmental, clinical, service use, hospital and temporal factors to all be associated with follow-up in this clinical population [6, 11, 18–22, 25]. Although the secondary exposure estimates may be biased, these associations warrant further examination in future work.

It is also important to note that the delivery of outpatient services has rapidly changed since March 2020 in response to the SARS-CoV-2 pandemic. The pandemic has generated a swift reconfiguration of service delivery to virtual care, including telepsychiatry [39] which has historically been underutilized [40]. Prior to March 2020, telepsychiatry had been identified as an avenue to improve poor rates of post-discharge care to people with mental health needs [40] and as a means to improve disparities in physician supply that exist between regions. Yet, challenges and barriers to the use of virtual care [41] may also be determined in part by geography (e.g., access to highspeed internet to successfully use a virtual platform, having a suitable location to privately meet with a provider virtually). Further examination of the use of virtual care in populations with SSD in relation to post-discharge follow-up should be undertaken.

### Strengths and limitations

To our knowledge, this is the first study that examines the impact of geospatial factors and distance on post-discharge quality of care among people with SSD. The focus on people diagnosed after first hospitalization is also a specific population that may be at a greater risk of negative outcomes. We also conducted sensitivity analyses to ensure our exposure of interest was not misclassified.

Although we excluded people with prior diagnoses of SSD, we were not able to account for people with affective psychoses (e.g., bipolar I disorder), who are at risk of diagnostic transition [42] and may be diagnosed with SSD during a hospitalization. This is important as people with affective psychoses are eligible for specialized mental health services like early psychosis intervention programs [43] that also provide services to people with SSD, which may impact service use post-discharge. We also restricted our regression analyses to people who had been discharged from a psychiatric bed (92.2% of all hospitalizations), as non-psychiatric beds do not report data to OMHRS which includes important clinical data that may confound the exposure-outcome relationship we were interested in.

We focused on physician services and could not account for contacts with non-physicians including nurses, case managers, social workers, and psychologists who all provide important aspects of care to this clinical population. Outpatient contacts with non-physician providers post-discharge would also meet the Ontario quality standard. Moreover, we were not able to ascertain whether a follow-up visit was arranged (which is the standard of care) but the person failed to attend, or if an appointment was never booked using the health administrative data available. Future studies may consider linking institutional and clinic-level data to examine these factors further. We were also unable to account for follow-up care which may have occurred outside of Ontario, which may be more likely to occur in regions bordering other provinces.

This study focused on one distance exposure, however other distance exposures may be important to consider in the context of post-discharge quality of care (e.g., the distance between a person’s residence to their outpatient provider). As noted, nearly all early psychosis intervention programs in the province are either hospital based or located near a hospital [14] and many people in this clinical population do not receive any outpatient care [6]. The exposure used for this study is a reasonable one to focus on considering the study’s emphasis on post-hospitalization discharge quality of care as well as the novel nature of this work in this clinical population and jurisdiction. This study highlights that it may be feasible to further examine other geospatial exposures in future work.

## Conclusions

Very few people who are diagnosed with SSD at the time of a first hospitalization received timely follow-up with a psychiatrist post-discharge in Ontario, Canada. Whether a person received follow-up care differs based on where they reside in the province. We did not find distance to be associated with psychiatric follow-up within 7 days post-discharge, however, increasing distance was independently associated with a reduction in the odds of psychiatric follow-up within 30 days post-discharge.

Our findings support the need for further work to evaluate the impact of geospatial factors on post-discharge quality of care and downstream outcomes in this clinical population. We need to consider developing, implementing, and evaluating interventions to improve post-discharge follow-up for people who have been diagnosed with SSD during a hospitalization.

## Supporting information

S1 Appendix Location of the 14 Local Health Integration Network (LHIN) regions in Ontario. (DOCX)

S2 Appendix Description of data sources. (DOCX)

S3 Appendix Covariate description, categorization and conceptual framework. (DOCX)

S4 Appendix Characteristics of the analytic cohort who received follow-up with psychiatrist within 7 days of hospitalization discharge vs. those who did not (n = 5,882). (DOCX)

S5 Appendix Sensitivity analyses. (DOCX)

S6 Appendix Dataset creation plan. (DOCX)
