# Supplementary material for: The role of geography and distance on physician follow-up after a first hospitalization with a diagnosis of a schizophrenia spectrum disorder: A retrospective population-based cohort study in Ontario, Canada
Source: PLoS One. 2023 Jun 16;18(6):e0287334. doi: 10.1371/journal.pone.0287334 (PMC10275454; doi:10.1371/journal.pone.0287334)
Supplement: S1 Appendix — (DOCX) [file pone.0287334.s001.docx]

**Appendix 1. Location of the 14 Local Health Integration Network (LHIN) regions in Ontario**


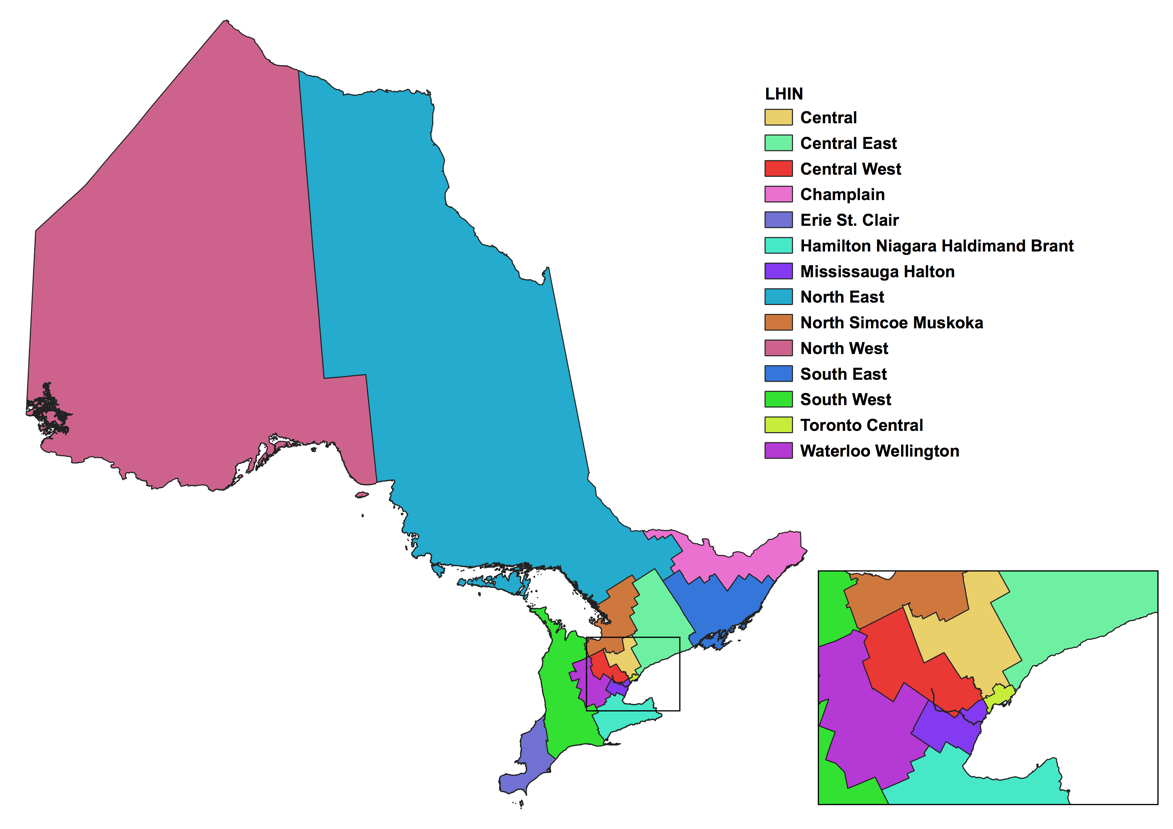


Note: This figure contains information licensed under the Open Government Licence – Ontario, https://www.ontario.ca/page/open-government-licence-ontario
