# Supplementary material for: The role of geography and distance on physician follow-up after a first hospitalization with a diagnosis of a schizophrenia spectrum disorder: A retrospective population-based cohort study in Ontario, Canada
Source: PLoS One. 2023 Jun 16;18(6):e0287334. doi: 10.1371/journal.pone.0287334 (PMC10275454; doi:10.1371/journal.pone.0287334)
Supplement: S2 Appendix — (DOCX) [file pone.0287334.s002.docx]

### **Description of data sources**

### Registered Persons Database (RPDB)

The RPDB contains data on basic demographics, location of residence, and mortality for all people in Ontario who are eligible for provincial health care coverage. These data are updated monthly and provided to ICES by the Ontario Ministry of Health and Long-term.^1^

### Ontario Mental Health Reporting System (OMHRS)

OMHRS contains data on all designated psychiatric beds for people aged 16 and older. Comprehensive data are collected using the Resident Assessment Instrument – Mental Health (RAI-MH)^2,3^ which includes information on mental and physical health, social supports, service use, outcome measures, and quality improvement indicators. Assessments are completed at the time of admission, discharge, and every three months during longer hospitalizations, as well as whenever there is a significant or unexpected change in a person’s clinical status.^4^ The Ontario MOHLTC mandated collection and reporting of these data to CIHI from October 2005 onwards.

### Discharge Abstract Database (DAD)

The DAD contains data on inpatient hospital discharges across Canada that are reported to the Canadian Institute for Health Information (CIHI). Facilities in all provinces and territories in Canada, except in Quebec, are required to report to CIHI.^5^ The DAD was initially developed in 1963 and is maintained by CIHI. It is based on abstracted data from the charts of patients after discharge, transfer or death and includes clinical, administrative and demographic data. ICES maintains DAD holdings from 1988 onwards for all acute care hospitalizations as well as historical inpatient mental health hospitalizations between 1998 to 2005. After 2005, reporting of adult inpatient mental health hospitalizations was transitioned to OMHRS. At the present time, information on child and adolescent mental health inpatient hospitalizations and psychiatric hospitalization in non-mental health units, i.e., intensive care units, general medicine units and other acute care services are part of the DAD. For mental health, hospitalization the DAD includes only minimal data on legal status, education level, marital status, and previous mental health hospitalizations – a smaller amount of information than is present in OMHRS. DAD and OMHRS hospitalizations do not overlap, however linkages at ICES create a single “episode of care” that accounts for transfers between beds which occurred between units reporting to either OMHRS or DAD. A typical scenario would be when someone presents to an emergency department (ED) and requires urgent medical attention in the context of a psychiatric presentation (e.g., a suicide attempt by overdose), there may be an admission to a general medical bed for medical care (reporting into DAD), followed by a transfer to a psychiatric bed for psychiatric care (reporting into OMHRS). Accounting for the transfers, this is treated as one single episode of care.

### National Ambulatory Care Reporting System (NACRS)

NACRS contains information on ED visits, day surgeries, and outpatient and community clinics^6^. Demographic, clinical, and administrative data are transmitted to CIHI from participating facilitates or from regional health authorities or ministries of health. ICES maintains data holdings on ED visits starting from July 2000.

### Ontario Health Insurance Plan (OHIP)

The OHIP claims database contains claims paid for by OHIP, the single-payer health insurance provider in Ontario. This database includes fee-for-service physician billings, as well as shadow billings for non-fee-for-service physicians and captures all specialist services and most family physician services other than those who practice in capitated or salaried practice models where patients are rostered to physicians and shadow billing does not occur. The database includes information on the specific physician identifiers, code for services provided, date of service and associated diagnosis. ICES receives OHIP claims data monthly from the Ministry of Health.

### ICES Physician Database (IPDB)

The IPDB contains data on physicians licensed to practice in Ontario, including information on specialization, training, and location. This database is created and maintained by ICES and based on data from the Ontario Physician Human Resource Data Centre (OPHRDC), the OHIP Corporate Provider Database (CPDB), and the OHIP database of physician billings.^7^

### Immigration, Refugees, and Citizenship Canada’s Permanent Resident Database (IRCC)

The Ontario portion of IRCC’s Permanent Resident Database includes data on immigration applications for people who initially landed in Ontario. These data contain information on country of citizenship, level of education, language, immigration class and landing date. The database includes data from 1985 onwards and does not include immigrants who reside in Ontario but originally landed in another province, nor immigrants who landed in Ontario and immediately moved to another province. These data are linked via a probabilistic linkage to the RPDB to obtain IKNs where possible, and linkage rates have improved over time from 70.5% in 1985 to 86.4% in 2012.^1^

### Ontario Marginalization Index (ON-Marg)

The ON-Marg is a validated index that provides area-level indicators of marginalization on four dimensions: i) material deprivation (area levels of poverty and inability to access and obtain basic material need), ii) residential instability (housing or family instability), iii) dependency (proportion of people who are not receiving income from paid employment or not being compensated for their work), and iv) ethnic concentration (proportion of people who are immigrants and/or identify as belonging to a visible minority group). These dimensional indices were derived from 18 variables from the Canadian census using principal component factor analysis of 42 possible census variables.^8^

###

### Postal Code Conversion File (PCCF)

The PCCF links six-character postal codes to standard geographic areas such as census tracts, subdivisions and dissemination areas.^9^ The PCCF also associates each postal code with a longitude and latitude coordinate to support further geographical analyses and mapping. The PCCF is jointly distributed and maintained by Statistics Canada and Canada Post Corporation. ICES maintains holdings of the PCCF beginning January 1994 and are updated with each new census.

### Canadian Census of Population

The Canadian Census of Population provides statistical information about the Canadian population. The 2016 Canadian Census is the most recent enumeration of Canadian residents and is conducted by Statistics Canada. Statistics Canada maintains estimates of population for provincial health regions.^10^

### Ontario Road Network (ORN): Road Net Element

The ORN is a database of over 250,000 km of roads and highways that is the authoritative source of road data for the Government of Ontario.^11^ It contains data on address information, road information and speed limits. It is published and distributed by the Government of Ontario, Ministry of Transportation.

**References**

1. Chiu M, Lebenbaum M, Lam K, et al. Describing the linkages of the immigration, refugees and citizenship Canada permanent resident data and vital statistics death registry to Ontario’s administrative health database. BMC Med Inform Decis Mak 2016;16(1):135.

2. Hirdes JP, Marhaba M, Smith TF, et al. Development of the Resident Assessment Instrument - Mental Health (RAI-MH). *Healthcare Quarterly*;4(2):https://www.longwoods.com/content/16756/healthcare-quarterly/development-of-the-resident-assessment-instrument-mental-health-rai-mh- (2000, accessed July 20, 2020).

3. Hirdes JP, Smith TF, Rabinowitz T, et al. The resident assessment instrument-mental health (RAI-MH): Inter-rater reliability and convergent validity. J Behav Health Serv Res 2002;29(4):419–432.

4. Ontario Mental Health Reporting System Metadata | CIHIhttps://www.cihi.ca/en/ontario-mental-health-reporting-system-metadata (accessed April 8, 2021).

5. Discharge Abstract Database metadata (DAD) | CIHIhttps://www.cihi.ca/en/discharge-abstract-database-metadata-dad (accessed April 8, 2021).

6. National Ambulatory Care Reporting System metadata (NACRS) | CIHIhttps://www.cihi.ca/en/national-ambulatory-care-reporting-system-metadata-nacrs (accessed April 8, 2021).

7. Slater M, Green ME, Shah B, et al. First Nations people with diabetes in Ontario: methods for a longitudinal population-based cohort study. CMAJ Open 2019;7(4):E680–E688.

8. Matheson F, Dunn JR, Smith KLW, et al. Development of the Canadian Marginalization Index: A New Tool for the Study of Inequality. Can J Public Health Rev Can Santee Publique 2012;103S12–S16.

9. Government of Canada SC. Postal Code OM Conversion Filehttps://www150.statcan.gc.ca/n1/en/catalogue/92-154-X (2017, accessed April 12, 2021).

10. Government of Canada SC. Estimates of population (2016 Census and administrative data), by age group and sex for July 1st, Canada, provinces, territories, health regions (2018 boundaries) and peer groupshttps://www150.statcan.gc.ca/t1/tbl1/en/tv.action?pid=1710013401 (2020, accessed April 25, 2021).

11. Ontario Road Network: Road Net Element - Ontario Data Cataloguehttps://data.ontario.ca/dataset/ontario-road-network-road-net-element (accessed July 16, 2020).
