# Supplementary material for: The role of geography and distance on physician follow-up after a first hospitalization with a diagnosis of a schizophrenia spectrum disorder: A retrospective population-based cohort study in Ontario, Canada
Source: PLoS One. 2023 Jun 16;18(6):e0287334. doi: 10.1371/journal.pone.0287334 (PMC10275454; doi:10.1371/journal.pone.0287334)
Supplement: S3 Appendix — (DOCX) [file pone.0287334.s003.docx]

### **Covariate description, categorization and conceptual framework**

### **Covariates**

Variables included in the analyses as potential confounding variables were identified based on i) the previous literature, ii) an a-priori conceptual framework, iii) a review of variables available in the ICES data holdings, and iv) clinical considerations and expertise. The variables were considered as secondary exposures and were grouped into relevant categories and identified as either sociodemographic, socioenvironmental, clinical, hospital or services use factors. A brief rationale as to why variables were included is also presented.

#### Sociodemographic Factors

##### Age

Age in years was calculated at the time of discharge and calculated based on date of birth from the RPDB and was used as a continuous variable. Age has previously been found to be associated with physician follow-up in people with SSD^1,2^ and is associated length of stay in hospital in people with SSD when travel distance is accounted for.^1,2^

##### Sex

Sex or gender is a factor that is commonly adjusted for in health services research and is associated with physician follow-up in people with a diagnosis of SSD.^2,4,5^ Biological sex was identified based on data from the RPDB and coded as male (M) or female (F).

##### Immigrant status

Previous Canadian work has found certain immigrant groups to have lower rates of health service utilization, and even lower rates of psychiatric service^6^ and other mental health service^7^ use. Specifically, Anderson^8^ has previously found immigrants from African and Caribbean countries to be less likely to receive psychiatric follow-up following hospitalization discharge in Ontario. These findings are consistent with data on follow-up and missed appointments in ethnoculturally diverse samples of people with SSD in other international settings.^4,5,9–11^ Previous work that has examined the role of distance on follow-up in other jurisdictions has found that membership in a majority group was associated with missing follow-up appointments after hospitalization discharge.^4^ In studies which do not account for distance, people of ethnic minority backgrounds have been found to be less likely to be referred to outpatient services following hospitalization discharge.^12^

People included in the cohort were categorizes into three categories based on IRCC data, as i) immigrants, ii) refugees and iii) non-immigrants. A separate refugee group was included, as previous work in Ontario has identified that refugees have an increased risk of developing an SSD independent of other risk factors,^1^ and may have different mobility and settlement patterns when compared to immigrants^13^ which may impact distance to and use of health services.

##### Housing stability

We included housing stability in our analysis, as stability of a person’s residence directly relates to the reliability of our main exposure measure, distance. Moreover, housing stability is a factor that impacts use of mental health services and other social services.^14^ Although previous work focusing on post-discharge follow-up in this clinical population has not specifically assessed this variable, previous data has found people with schizophrenia who are homeless are less likely to receive physician follow-up after hospitalization discharge.^15^ A measure of housing stability was obtained from OMHRS and is based on a RAI-MH assessment item that assesses whether a person’s last residence is considered temporary or not. Temporary residence is defined as a place of residence that a person has lived for less than 30 days and plans to move within 30 days (e.g., shelter, hostel)^16^. We coded this variable as 0 if the person’s last residence was not considered temporary, and 1 if the person’s last residence was considered temporary.

##### Living arrangements

We used a person’s living arrangements as an indicator of social support. Social support is a factor that is associated with both psychiatric and general medical service use in populations with mental health issues.^17,18^ In our literature review, family support was found to be associated with follow-up.^4,19^ Considering the age of people included in this cohort, living arrangements may be a more relevant indicator when compared to general marital status, as it also includes living arrangements with a spouse. People were categorized based on the RAI-MH assessment item from OMHRS based on the following options: 1 = Lived Alone, 2 = Lived with spouse only, 3 = Lived with spouse and other, 4 = Lived with child (not spouse), 5 = Lived with other (s) (not spouse or children), and 6 = Lived in group setting with non-relative. The variable was dichotomized and coded as either 1 (lived alone), or 0 (did not live alone) if any other options were selected.

#### Socioenvironmental

##### LHIN region

Previous work by *Anderson and Kurdyak* in this clinical population has accounted for clustering by health administrative region at the LHIN level.^1^ In our literature review, previous studies have found physician supply in a region to be associated with follow-up.^20^ Moreover, previous studies have also found disparities in the supply of psychiatrists across LHINs,^21^ and that service use, including likelihood of access to a psychiatrist, varies based on LHIN and may relate to regional variation in the supply of services and practice pattern differences.^22^ We assigned all people to one of the 14 LHIN regions based on RPDB data at the time of discharge. The regions included: 1) Erie St. Clair 2) South West 3) Waterloo Wellington 4) Hamilton Niagara Haldimand Brant 5) Central West 6) Mississauga Halton 7) Toronto Central 8) Central 9) Central East 10) South East 11) Champlain 12) North Simcoe Muskoka 13) North East and 14) North West.

##### Sub-LHIN region

Sub-LHINs are subdivisions of the 14 LHINs. For the purpose of multilevel modeling we used the sub-LHIN level, rather than region, as a random intercept, considering i) a greater number of groups is more informative and preferable in multilevel models, as a smaller number of groups may lead to biased estimates^23^ and ii) based on the size of the sub-LHINs, people residing in the same sub-LHIN are likely exposed to more similar conditions and have similar access to care when compared to people residing in the larger LHIN region. We assigned people to one of the 97 primary sub-LHINs based on RPDB data at the time of discharge.

##### Rural residence

Living in a rural area is associated with lower rates of post-discharge follow-up in previous studies in Ontario in this clinical population,^1^ as well other studies which included samples of people with schizophrenia,^2,11^ and studies from other jurisdictions where distance from hospital on follow-up has been studied.^4^ Rural residence was defined based on the Rurality Index of Ontario (RIO) score for each person based on the location of their residence, obtained from the RPDB. A RIO score is based on measures of community population, population density, travel time to nearest basic referral center and travel time to nearest advanced referral center for a given area^24^. Higher RIO scores reflect a higher degree of rurality and RIO scores of 40 or above are characterized as rural.^25^ People residing in non-rural areas were coded as 0 and people living in rural areas were coded as 1.

##### Area-level marginalization

Area-level marginalization is associated with the risk of developing a SSD in Ontario.^26^ From a service use perspective, people with non-psychotic mental disorder who live in more deprived areas in the province have been found to use more mental health services.^27^ In samples of people with psychosis in Ontario, neighbourhood-level marginalization has been shown to be associated with service use pathways^28^ and rates of inpatient hospitalization.^27^ In previous population based cohorts looking at post-discharge follow-up in people with schizophrenia in other jurisdictions, area-level poverty was associated with follow-up.^20^ The impact of area level-level marginalization beyond the dimension of material deprivation^1^ on service use following hospitalization discharge has not yet been examined in this clinical population in Ontario.

Exposure to area-level marginalization was ascertained using the Ontario Marginalization Index (ON-Marg). The ON-Marg is a validated index of marginalization and includes four dimensions: i) material deprivation (which is an indicator of area levels of poverty and inability to access and obtain basic material needs), ii) residential instability (which is an indicator of housing or family instability), iii) dependency (which is an indicator of the proportion of people who are not receiving income from paid employment or not being compensated for their work), and iv) ethnic concentration (which is an indicator of the proportion of people who are immigrants and/or identify as belonging to a visible minority group). These dimensional indices were derived from 18 variables from the Canadian census using principal component factor analysis of 42 possible census variables^29^. The index can be used at different levels of census geographies. To minimize measurement error we used the index at the census dissemination area (DA)-level, the smallest standard geographic area for which all census data are disseminated,^30^ and the smallest spatial area available for use with the ON-Marg.^31^ Each person was assigned to a quintile for each of the 4 marginalization dimensions based on the DA they reside in, ranging from 1 (lowest level of marginalization) to 5 (highest level of marginalization).

Considering individual-level data socioeconomic data are not yet available in any of the linked ICES data holdings, the DA-level ON-Marg quintiles can also be used as a proxy for socioeconomic status.^31^

#### Clinical

##### Diagnosis

We included a spectrum of people with SSD in the cohort as many people with a diagnosis of a psychosis NOS will ultimately be diagnosed with schizophrenia^32^ and tend to have similar health services use trajectories to those with a diagnosis of schizophrenia or schizoaffective disorder, and may even be at greater risk of hospitalization.^33^ It is not entirely clear if this remains the case when other factors, including geospatial factors, are taken into account in this clinical population in Ontario. Previous studies which have examined follow-up in cohorts including people with schizophrenia and serious mental illness have found an association specific diagnoses and follow-up.^5,20^ We coded people as having a diagnosis of either Schizophrenia or Schizoaffective Disorder (DSM-IV/ICD-9: 295, or ICD-10-CA: F20, F25) or ii) Psychosis NOS (DSM-IV/ICD-9: 298, or ICD-10-CA: F29) based on diagnostic codes obtained from DAD and OMHRS at the time of hospitalization discharge.

##### Problematic substance use

A history of substance use is associated with a lower likelihood of outpatient follow-up post discharge^20^ and is associated with an increased risk of rehospitalization in a previous study assessing the role of distance to hospital in a cohort of people with schizophrenia.^34^ The RAI-MH assessment includes data on substance use history and provides a summary measure of problematic substance use. This measure captures a range of substances that a person may be taking or has used in the past, and includes prescription drug and over-the counter-medication misuse. This measure has been found to have sensitivity of 97.3% and specificity of 68.3%.^35^ We coded this variable as i) current problems with substance use, or ii) no current problems with substance use.

##### Length of stay

Previous studies have found that people with schizophrenia who are hospitalized further away from home have a longer length of stay (LOS),^3^ and LOS is associated with follow-up after hospitalization discharge in samples of people with SSD.^5^ LOS is also associated with post-discharge care coordination during an acute hospitalization.^36^ LOS was calculated as the number of days between the hospitalization discharge date and admission date for the entire episode of care based on OMHRS and DAD data.

##### Positive Symptom Scale

Smaller studies which have examined missed appointments in samples of people with SSD have found symptom severity to be associated with missed follow-up care.^4,9^ It is likely that larger studies using health administrative data have not had data available on symptom severity. The long form of the Positive Symptom Scale (PSS) was used in the current study as a measure of symptom severity, which is most relevant in a clinical population with SSD. We did not include measures of negative symptoms or affective symptoms, as clinically it can be challenging to differentiate primary negative symptoms as well as affective symptoms from secondary negative symptoms that may be related to other factors (including but not limited to residual positive symptoms and medication side effects).^37^

The PSS is a RAI-MH measure available in OMHRS. The PSS has been validated against the Positive and Negative Syndrome Scale (PANSS).^38^ The PSS is based on the presence of hallucinations, delusions, abnormal thought process/form, inflated self-worth, hyperarousal, pressured speech, and abnormal/unusual movements. We used PSS scores from the RAI discharge assessment. Scores on the PSS range from 0 to 24, and higher scores indicate a greater severity of positive symptoms of psychosis.

##### Involuntary Admission

To our knowledge, involuntary admission at the index hospitalization has not yet been studied in relation to follow-up in this clinical population. Although involuntary status has been used in some studies as an indicator of symptom and illness severity^15^ which is associated with follow-up care,^4,9^ it may be most appropriate to use this measure as an indicator of risk. Presentations with a greater degree of risk to oneself and others warrant close and follow-up and intervention. Previous studies in Ontario have found an elevated mortality in this clinical subgroup, particularly within the 5 year period after initial involuntary hospitalization, and there have been calls for follow-up to reduce mortality.^39^

An involuntary admission occurs when a person is detained in a facility without their consent. The Ontario Mental Health Act defines an involuntary patient as detainment in a psychiatric facility under a certificate of involuntary admission (Form 3), a certificate of renewal (Form 4) or a certificate of continuation (Form 4a).^40^ A person may also be admitted to hospital while they are detained under an application for psychiatric assessment (Form 1) or as an informal patient where they may be admitted with the consent of another person under the Health Care Consent Act.

We used OMHRS data at the time of hospitalization admission to ascertain whether a person was admitted to hospital voluntarily or involuntarily. Inpatient status at the time of admission was coded in OMHRS as either 1) Application for psychiatric assessment, 2) Voluntary, 3) Informal, 4) Involuntary and 5) Forensic. All Forensic admissions were excluded from the cohort. We created a dichotomous variable and coded all categories other than the OMHRS voluntary category as an involuntary admission.

##### Insight

Insight has been found to be associated with post-discharge follow-up care in a small study of people with SSD after an initial hospitalization.^19^ Insight during early phases of a SSD is associated with treatment adherence and general clinical course.^41^ Insight is also correlated with capacity to consent to treatment^42^ and downstream functional capacity,^43^ which have implications for the role of Community Treatment Orders in this population^44^ which may mandate treatment and follow-up if specific criteria are met.

We used RAI-MH assessment data from OMHRS at the time of hospitalization discharge. The RAI-MH assesses insight into mental health as being either full, i.e., the person recognizes that a problem exists and appears to understand the problem or that he or she needs treatment, limited (i.e., the person acknowledges the problem but may not be able to identify causative factors) or no insight (i.e., the person appears to have no awareness of difficulties or the presence of a mental health problem). This variable was codes as 0 for full insight, 1 for limited insight and 2 for no insight.

#### Hospital

Hospital level factors can impact continuity of care and follow-up.^45^ Different hospital settings may have differential access to specific outpatient services and may also be related to physician supply in an area. For the purpose of this study, we categorized whether the facility a person was discharged from was an academic hospital based on the institution number which was linked to data held on Ontario health care institutions funded by the Ministry of Health and Long-Term Care. Although this variable has not specifically been studied in the literature in this clinical population, the structure of “community” vs. “academic” hospitals may impact follow-up care and warrants further consideration.

#### Service Use

Previous service contacts are important to consider, as people who have previous contact with either psychiatrists or FPs for any mental health reason would be more likely to connect with these services following hospitalization discharge. Moreover, people who have previously been hospitalized or presented to an ED for mental health reasons may routinely be referred to outpatient services. ^20,46–48^ The previous literature which has examined follow-up in samples of people with a SSD or included people with a SSD has found prior service use to be associated with follow-up post-discharge.^1,5,10,11,19^ We constructed a number of binary indicators of prior service use relevant to this clinical population, described below.

##### Previous Contact with a Psychiatrist

We identified any visit with a psychiatrist based on OHIP billing claims in the year prior to hospitalization and created a dichotomous variable indicating whether there was any contact with an outpatient psychiatrist in the year prior to index hospitalization.

##### Previous Contact with a Family Physician

Contact with a FP was defined as any visit to a FP for a mental health reason within 1 year prior to the index hospitalization for any mental health reason, and both were used as a dichotomous variable. FP visits were identified using OHIP billing claims using a validated algorithm that identifies mental health service provision in primary care settings.^49^

##### Recent Emergency Department Contact

Any ED visits for any mental health reasons were identified from NACRS using relevant ICD codes in the year prior to hospitalization. We created a dichotomous variable to indicate whether any ED visits for mental health reasons occurred in the year prior to index hospitalization.

##### Recent Hospitalization for a Mental Health Reason

Any hospitalization for a mental health reason other than SSD was identified based on OMHRS and DAD data using relevant ICD and DSM codes in the year prior to the index hospitalization, and this was used as a dichotomous variable.

#### Year of Discharge

Previous work looking at follow-up in this clinical population in the same jurisdiction has found the year of discharge to be associated with follow-up^1^. Previous work that has looked at the association between distance and re-hospitalization has also attempted to account for an earthquake that occurred during the study period.^3^ We accounted for potential changes to the structure and funding of mental health services across the province during the study period based on year of hospitalization discharge. Year of discharge was recorded based on discharge date from either OMHRS or DAD.

Conceptual framework of factors impacting follow-up after hospitalization discharge after an incident diagnosis of a schizophrenia spectrum disorder SSD


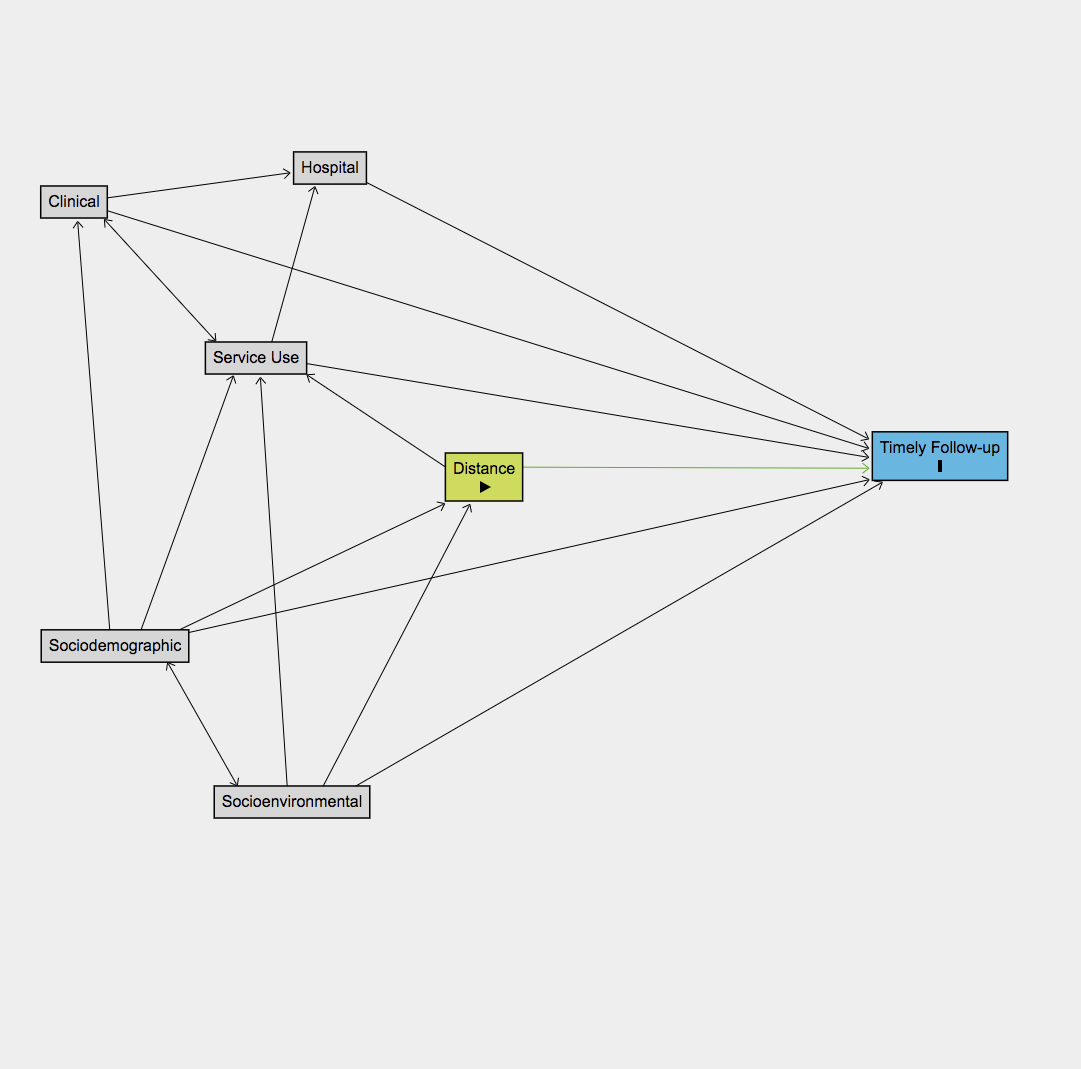


Health administrative region

**Notes:**

Grey box = factors adjusted for

Green box = exposure variable

Blue box = outcome

Green line = relationship of interest

Orange dash box = clustering accounted for by multilevel framework

**References**

1. Anderson KK, Kurdyak P. Factors Associated with Timely Physician Follow-up after a First Diagnosis of Psychotic Disorder. Can J Psychiatry Rev Can Psychiatr 2017;62(4):268–277.

2. Kurdyak P, Vigod SN, Newman A, et al. Impact of Physician Follow-Up Care on Psychiatric Readmission Rates in a Population-Based Sample of Patients With Schizophrenia. Psychiatr Serv 2018;69(1):61–68.

3. Tseng K-C, Hemenway D, Kawachi I, et al. Travel Distance and the Use of Inpatient Care among Patients with Schizophrenia. Adm Policy Ment Health 2008;35(5):346–356.

4. Nwefoh E, Aguocha CM, Achor J, et al. Missed post-hospitalisation clinic appointment in a psychiatric hospital in Southeast Nigeria. Ment Health Relig Cult 2018;21(6):564–577.

5. Marcus SC, Chuang C-C, Ng-Mak DS, et al. Outpatient Follow-Up Care and Risk of Hospital Readmission in Schizophrenia and Bipolar Disorder. Psychiatr Serv Wash DC 2017;68(12):1239–1246.

6. Chen AW, Kazanjian A. Rate of Mental Health Service Utilization by Chinese Immigrants in British Columbia. Can J Public Health 2005;96(1):49–51.

7. Kirmayer LJ, Weinfeld M, Burgos G, et al. Use of Health Care Services for Psychological Distress by Immigrants in an Urban Multicultural Milieu. Can J Psychiatry 2007;52(5):295–304.

8. Anderson KK. Ethnic differences in physician follow-up after a first diagnosis of psychotic disorder. Schizophr Res 2018;193463–464.

9. Adeponle AB, Baduku AS, Adelekan ML, et al. Prospective Study of Psychiatric Follow-up Default and Medication Compliance after Discharge at a Psychiatric Hospital in Nigeria. Community Ment Health J 2009;45(1):19–25.

10. Marino L, Wissow LS, Davis M, et al. Predictors of outpatient mental health clinic follow-up after hospitalization among Medicaid-enrolled young adults. Early Interv Psychiatry 2016;10(6):468–475.

11. Carson NJ, Vesper A, Chen C, et al. Quality of Follow-Up After Hospitalization for Mental Illness Among Patients From Racial-Ethnic Minority Groups. Psychiatr Serv 2014;65(7):888–896.

12. Thompson EE, Neighbors HW, Munday C, et al. Length of stay, referral to aftercare, and rehospitalization among psychiatric inpatients. Psychiatr Serv Wash DC 2003;54(9):1271–1276.

13. Government of Canada SC. Are Refugees More Likely to Leave Initial Destinations than Economic Immigrants? Recent Evidence from Canadian Longitudinal Administrative Datahttps://www150.statcan.gc.ca/n1/pub/11f0019m/11f0019m2020004-eng.htm (2020, accessed April 14, 2021).

14. Kerman N, Sylvestre J, Aubry T, et al. The effects of housing stability on service use among homeless adults with mental illness in a randomized controlled trial of housing first. BMC Health Serv Res 2018;18(1):190.

15. Laliberté V, Stergiopoulos V, Jacob B, et al. Homelessness at discharge and its impact on psychiatric readmission and physician follow-up: a population-based cohort study. Epidemiol Psychiatr Sci;29. Epub ahead of print ed 2020. DOI: 10.1017/S2045796019000052.

16. Canadian Institute for Health Information. Ontario Mental Health Reporting System Resource Manual, 2013–2014. 2013.

17. Maulik PK, Eaton WW, Bradshaw CP. The Role of Social Network and Support in Mental Health Service Use: Findings From the Baltimore ECA Study. Psychiatr Serv 2009;60(9):1222–1229.

18. Albert M, Becker T, Mccrone P, et al. Social Networks and Mental Health Service Utilisation - a Literature Review. Int J Soc Psychiatry 1998;44(4):248–266.

19. Compton MT. Barriers to Initial Outpatient Treatment Engagement Following First Hospitalization for a First Episode of Nonaffective Psychosis: A Descriptive Case Series. J Psychiatr Pract 2005;11(1):62–69.

20. Olfson M, Marcus SC, Doshi JA. Continuity of Care After Inpatient Discharge of Patients With Schizophrenia in the Medicaid Program: A Retrospective Longitudinal Cohort Analysis. J Clin Psychiatry 2010;71(7):0–0.

21. Kurdyak P, Stukel TA, Goldbloom D, et al. Universal coverage without universal access: a study of psychiatrist supply and practice patterns in Ontario. Open Med 2014;8(3):e87–e99.

22. Martin L, Hirdes JP. Mental health needs and service use in Ontario. Healthc Manage Forum 2009;22(1):40–46.

23. Maas CJM, Hox JJ. Sufficient Sample Sizes for Multilevel Modeling. Methodol Eur J Res Methods Behav Soc Sci 2005;1(3):86–92.

24. Kralji B. Measuring Rurality - RIO2008 BASIC: Methodology and Results. Toronto, ON: Ontario Medical Association Economics Department;http://www.eriestclairlhin.on.ca/Page.aspx?id=11606 (2009, accessed April 15, 2021).

25. Glazier R, Zagorski B, Rayner J. Comparison of primary care models in Ontario by demographics, case mix and emergency department use, 2008/09 to 2009/10. Toronto, ON: Institute for Clinical Evaluative Sciences;https://www.ices.on.ca/flip-publication/comparison-of-primary-care-models-in-ontario-by-demographics/files/assets/basic-html/page15.html (2012, accessed April 15, 2021).

26. Rotenberg M, Tuck A, Anderson KK, et al. The Incidence of Psychotic Disorders and Area-level Marginalization in Ontario, Canada: A Population-based Retrospective Cohort Study. Can J Psychiatry 2021;07067437211011852.

27. Durbin A, Moineddin R, Lin E, et al. Examining the relationship between neighbourhood deprivation and mental health service use of immigrants in Ontario, Canada: a cross-sectional study. BMJ Open 2015;5(3):e006690.

28. Rotenberg M, Tuck A, Ptashny R, et al. The role of ethnicity in pathways to emergency psychiatric services for clients with psychosis. BMC Psychiatry 2017;17(1):137.

29. Matheson F, Dunn JR, Smith KLW, et al. Development of the Canadian Marginalization Index: A New Tool for the Study of Inequality. Can J Public Health Rev Can Santee Publique 2012;103S12–S16.

30. Dissemination area: Detailed definitionhttps://www150.statcan.gc.ca/n1/pub/92-195-x/2011001/geo/da-ad/def-eng.htm (accessed April 15, 2021).

31. Matheson F, van Ingen T. 2016 Ontario marginalization index: user guide. Toronto, ON: St. Michael’s Hospital; 2018.

32. Murrie B, Lappin J, Large M, et al. Transition of Substance-Induced, Brief, and Atypical Psychoses to Schizophrenia: A Systematic Review and Meta-analysis. Schizophr Bull 2020;46(3):505–516.

33. Rodrigues R, MacDougall AG, Zou G, et al. Involuntary hospitalization among young people with early psychosis: A population-based study using health administrative data. Schizophr Res 2019;208276–284.

34. Hudson CG. Five-year rehospitalization experience of a state-wide cohort of persons with schizophrenia. Soc Psychiatry Psychiatr Epidemiol 2019;54(7):861–870.

35. Martin L, Hirdes JP, Morris JN, et al. Validating the Mental Health Assessment Protocols (MHAPs) in the Resident Assessment Instrument Mental Health (RAI-MH). J Psychiatr Ment Health Nurs 2009;16(7):646–653.

36. Nakanishi M, Niimura J, Tanoue M, et al. Association between length of hospital stay and implementation of discharge planning in acute psychiatric inpatients in Japan. Int J Ment Health Syst;9. Epub ahead of print May 30, 2015. DOI: 10.1186/s13033-015-0015-9.

37. Correll CU, Schooler NR. Negative Symptoms in Schizophrenia: A Review and Clinical Guide for Recognition, Assessment, and Treatment. Neuropsychiatr Dis Treat 2020;16519–534.

38. Scales: Status and Outcome Measures – interRAIhttps://www.interrai.org/scales.html (accessed April 15, 2021).

39. Kurdyak P, Mallia E, de Oliveira C, et al. Mortality After the First Diagnosis of Schizophrenia-Spectrum Disorders: A Population-based Retrospective Cohort Study. Schizophr Bull 2021;47(3):864–874.

40. Mental Health Act, R.S.O. 1990, c. M.7.https://www.ontario.ca/laws/view (accessed April 27, 2021).

41. Segarra R, Ojeda N, Peña J, et al. Longitudinal changes of insight in first episode psychosis and its relation to clinical symptoms, treatment adherence and global functioning: One-year follow-up from the Eiffel study. Eur Psychiatry 2012;27(1):43–49.

42. Raffard S, Fond G, Brittner M, et al. Cognitive insight as an indicator of competence to consent to treatment in schizophrenia. Schizophr Res 2013;144(1):118–121.

43. Roseman AS, Kasckow J, Fellows I, et al. Insight, quality of life, and functional capacity in middle-aged and older adults with schizophrenia. Int J Geriatr Psychiatry 2008;23(7):760–765.

44. Levy E, Mustafa S, Naveed K, et al. Effectiveness of Community Treatment Order in Patients with a First Episode of Psychosis: A Mirror-Image Study. Can J Psychiatry Rev Can Psychiatr 2018;63(11):766–773.

45. Fontanella CA, Hiance-Steelesmith DL, Bridge JA, et al. Factors Associated With Timely Follow-Up Care After Psychiatric Hospitalization for Youths With Mood Disorders. Psychiatr Serv Wash DC 2016;67(3):324–331.

46. Vukmir RB, Kremen R, Dehart DA, et al. Compliance with emergency department patient referral. Am J Emerg Med 1992;10(5):413–417.

47. Dobscha SK, Delucchi K, Young ML. Adherence with Referrals for Outpatient Follow-Up from a VA Psychiatric Emergency Room. Community Ment Health J 1999;35(5):451–458.

48. Olfson M, Mechanic D, Boyer CA, et al. Linking Inpatients With Schizophrenia to Outpatient Care. Psychiatr Serv 1998;49(7):911–917.

49. Steele LS, Glazier RH, Lin E, et al. Using Administrative Data to Measure Ambulatory Mental Health Service Provision in Primary Care. Med Care 2004;42(10):960–965.
