# Supplementary material for: The role of geography and distance on physician follow-up after a first hospitalization with a diagnosis of a schizophrenia spectrum disorder: A retrospective population-based cohort study in Ontario, Canada
Source: PLoS One. 2023 Jun 16;18(6):e0287334. doi: 10.1371/journal.pone.0287334 (PMC10275454; doi:10.1371/journal.pone.0287334)
Supplement: S4 Appendix — (DOCX) [file pone.0287334.s004.docx]

Appendix 4. Characteristics of the analytic cohort who received follow-up with psychiatrist within 7 days of hospitalization discharge vs. those who did not (n = 5,882)

| **Variable** |  | **Follow-up with a psychiatrist within 7 days of discharge** |  | **No psychiatrist follow-up within 7 days of discharge** | **Standardized difference** |
| --- | --- | --- | --- | --- | --- |
| n (%) * |  | 815 (14) |  | 5067 (86) |  |
| Distance (km)^a^ |  |  |  |  |  |
| Mean (SD) |  | 22.6 (54.4) |  | 31.5 (80.2) |  |
| Median [IQR] |  | 8.0 [3.9, 18.4] |  | 9.7 [3.9, 24.1] | **0.13** |
| Age (year) |  |  |  |  |  |
| Mean (SD) |  | 25.5(6.3) |  | 25.8 (6.4) |  |
| Male |  | 511 (62.7) |  | 3411 (67.3) | 0.01 |
| Immigration history |  | |  | | 0.02 |
| General Population |  | 673 (82.6) |  | 4208 (83.0) |  |
| Immigration |  | 108 (13.3) |  | 637 (12.6) |  |
| Refugee |  | 34 (4.2) |  | 222 (4.4) |  |
| Housing stability ^b^ |  | 214 (26.4) |  | 1311 (26.1) | 0.01 |
| Lives alone ^c^ |  | 671 (82.6) |  | 4039 (80.3) | 0.06 |
| Rural residence ^d^ |  | 48 (5.9) |  | 570 (11.3) | **0.19** |
| Area-level marginalization |  |  |  |  |  |
| Dependency ^e^ |  | |  | | 0.10 |
| Q1 (low) |  | 226 (28.2) |  | 1320 (26.8) |  |
| Q2 |  | 181 (22.6) |  | 1058 (21.5) |  |
| Q3 |  | 158 (19.7) |  | 882 (17.9) |  |
| Q4 |  | 121 (15.1) |  | 803 (16.3) |  |
| Q5 (high) |  | 115 (14.4) |  | 863 (17.5) |  |
| Deprivation ^e^ |  | |  | | 0.10 |
| Q1 (low) |  | 141 (17.6) |  | 720 (14.6) |  |
| Q2 |  | 133 (16.6) |  | 745 (15.1) |  |
| Q3 |  | 145 (18.1) |  | 915 (18.6) |  |
| Q4 |  | 155 (19.4) |  | 1066 (21.6) |  |
| Q5 (high) |  | 227 (28.3) |  | 1480 (30.0) |  |
| Ethnic concentration ^e^ |  | |  | | **0.27** |
| Q1 (low) |  | 80 (10.0) |  | 798 (16.2) |  |
| Q2 |  | 96 (12.0) |  | 824 (16.7) |  |
| Q3 |  | 146 (18.2) |  | 817 (16.6) |  |
| Q4 |  | 212 (26.5) |  | 940 (19.1) |  |
| Q5 (high) |  | 267 (33.3) |  | 1547 (31.4) |  |
| Residential instability ^e^ |  | |  | | 0.09 |
| Q1 (low) |  | 134 (16.7) |  | 826 (16.8) |  |
| Q2 |  | 114 (14.2) |  | 805 (16.3) |  |
| Q3 |  | 134 (16.7) |  | 816 (16.6) |  |
| Q4 |  | 159 (19.9) |  | 1051 (21.3) |  |
| Q5 (high) |  | 260 (32.5) |  | 1428 (29.0) |  |
| Psychosis NOS |  | 472 (57.9) |  | 2977 (58.8) | 0.02 |
| Voluntary admission |  | 141 (17.3) |  | 566 (11.2) | **0.18** |
| Insight |  | |  | | 0.08 |
| Full |  | 134 (16.4) |  | 700 (13.8) |  |
| Limited |  | 481 (59.0) |  | 3013 (59.5) |  |
| None |  | 200 (24.5) |  | 1354 (26.7) |  |
| Problematic substance use ^f^ |  | 374 (46.0) |  | 2708 (53.7) | **0.15** |
| PSS (score) ^f^ |  |  |  |  |  |
| Mean (SD) |  | 2.2 (3.2) |  | 2.3 (3.3) |  |
| Median [IQR] |  | 0.0 [0.0, 4.00] |  | 0.0 [0.0, 4.0] | 0.02 |
| Length of stay (days) |  |  |  |  |  |
| Mean (SD) |  | 22.1 (15.7) |  | 17.9 (14.0) |  |
| Median [IQR] |  | 16.0 [10.0, 28.0] |  | 14.0 [9.0, 22.0] | **0.21** |
| Teaching hospital |  | 339 (41.6) |  | 1414 (27.9) | **0.29** |
| Psychiatrist contact (in prev. year) |  | 303 (37.2) |  | 1118 (22.1) | **0.16** |
| Mental health contact with FP (in prev. year) |  | 404 (49.6) |  | 2206 (43.5) | **0.12** |
| Mental health ED visit (in prev. year) |  | 362 (44.4) |  | 2258 (44.6) | 0.003 |
| Mental health hospitalization (in prev. year) |  | 74 (9.1) |  | 462 (9.1) | 0.001 |
|  |  |  |  |  |  |
|  |  |  |  |  |  |

Notes: * n (%) unless otherwise specified, ^a^ 46 (0.8%) missing observations, ^b^ 42 (0.7%) missing observations, ^c^ 41 (0.7%) missing observations, ^d^ 48 (0.8%) missing observations, ^e^ 155 (2.6%) missing observations, ^f^ 28 (0.5%) missing observations
