# Supplementary material for: The role of geography and distance on physician follow-up after a first hospitalization with a diagnosis of a schizophrenia spectrum disorder: A retrospective population-based cohort study in Ontario, Canada
Source: PLoS One. 2023 Jun 16;18(6):e0287334. doi: 10.1371/journal.pone.0287334 (PMC10275454; doi:10.1371/journal.pone.0287334)
Supplement: S5 Appendix — (DOCX) [file pone.0287334.s005.docx]

## Sensitivity analyses

We conducted several sensitivity analyses focusing on the primary exposure of interest distance.

### Alternative exposure definitions

Although the majority of studies investigating distance decay effects on health service use a Euclidean distance, it is unlikely that actual travel occurs in a straight-line.^1^ Fully adjusted model estimates were compared with alternative ways of measuring the primary exposure of interest by calculating distance from discharging hospital to a person’s residence via i) drive time and ii) driving distance.

To estimate the travel distance and drive time from a person's residence to the discharging hospital a GIS method of network analysis was used. This method was carried out using ArcGIS version 10.2 and the "Network Analyst” module. Locations of patients and hospitals were defined based on their corresponding postal codes and using PCCF. The GIS road network data were available from the open source Ontario Road Network (ORN): Road Net Element. Output estimates were provided in minutes and metres which was subsequently converted to kilometers.

When we fit fully adjusted models for primary and secondary outcomes with alternative definitions of the primary exposure of interest – specifically, driving distance and driving time instead of Euclidian distance – we found no substantive change in effect estimates or statistical significance, when compared to the models with Euclidian distance. This suggests that defining distance using Euclidian distance is comparable to alternative definitions based on driving distance and drive time.

### Restricted samples

Some people with SSD may be highly mobile, and previous Canadian literature from Quebec suggests that one third of people may make a major move from one health region to another within six years following diagnosis of schizophrenia.^2^ Mobility may result in a misclassification of postal codes and the exposure of interest.

There is also risk that people who have traveled extreme distances between location of hospitalization and their residence may not have the most up-to-date residential information that is captured in the linked health administrative data. These extreme cases can also be restricted from the sample.

The analytic cohort cohort was restricted in the following ways: i) to people who were deemed to have stable housing, ii) to people whose residence was below the 90^th^ percentile distance from place of hospitalization (less than 63.4 km), and iii) to people whose residence was below the 75^th^ percentile distance from place of hospitalization (less than 23.3 km). In the restricted sample of people who had stable housing distance ranged between 0 to 1345.50 km with a median distance of 9.34 km (IQR: 3.92 - 22.88).

Model estimates based on the restricted samples and the analytic sample were compared to assess for potential misclassification of the exposure.

When a fully adjusted model was fit with the outcome of follow-up with a psychiatrist within 7 days of discharge in a sample restricted to only to people with stable housing, there was no change in the model estimate for distance. In the fully adjusted models fit with the outcome of follow-up with a psychiatrist within 7 days of discharge in samples restricted to observations with distances below the 90^th^ percentile and below the 75^th^ percentiles, there was no change in the estimates for distance.

When a fully adjusted model was fit with the secondary outcome of follow-up with a psychiatrist within 30 days of discharge in a sample restricted to people with stable housing, there was no change in the estimate for distance. In the fully adjusted model fit with the outcome of follow-up with a psychiatrist within 30 days of discharge in samples restricted to observations with distances below the 90^th^ percentile and below the 75^th^ percentiles the association between distance and follow-up was no longer present. The loss of significance in the restricted distance samples support the finding that distance, particularly people who live longer distances from hospital, are recipients of poorer quality of post-discharge care.

There were no changes to the distance estimates based on the restricted samples when models were fit with the secondary outcomes of any physician follow-up with both 7 and 30 days of hospitalization discharge.

**References**

1. Kelly C, Hulme C, Farragher T, et al. Are differences in travel time or distance to healthcare for adults in global north countries associated with an impact on health outcomes? A systematic review. BMJ Open 2016;6(11):e013059.

2. Ngamini Ngui A, Cohen AA, Courteau J, et al. Does elapsed time between first diagnosis of schizophrenia and migration between health territories vary by place of residence? A survival analysis approach. Health Place 2013;2066–74.
