# Supplementary material for: The role of geography and distance on physician follow-up after a first hospitalization with a diagnosis of a schizophrenia spectrum disorder: A retrospective population-based cohort study in Ontario, Canada
Source: PLoS One. 2023 Jun 16;18(6):e0287334. doi: 10.1371/journal.pone.0287334 (PMC10275454; doi:10.1371/journal.pone.0287334)
Supplement: S6 Appendix — (DOCX) [file pone.0287334.s006.docx]

Dataset creation plan

| Project Initiation  This Section must be Completed Prior to Project Dataset(s) Creation | | | | | | |
| --- | --- | --- | --- | --- | --- | --- |
| **Project Title:** | Physician follow up after a first hospitalization for schizophrenia | | | | | |
| **Project TRIM number:** | 2021 0900 300 061 | | | | | |
| **Research Program:** | MHA | | | | | |
| **Site:** | ICES Central | | | | | |
| **Project Objectives:** | *Insert Project Objectives as listed in the approved ICES Project PIA* | | | | | |
|  | - To estimate the rate of physician follow-up after a first hospitalization for schizophrenia in Ontario. - To examine the impact of geography on likelihood to receive timely physician follow-up after a first hospitalization for schizophrenia - Explore how readmission rates after a first hospitalization for schizophrenia differ based on geography and follow-up | | | | | |
| **ICES Project PIA Initial Approval Date:** | *The ICES Employee or agent who is responsible for creating the Project Dataset(s) is responsible for ensuring there is an approved ICES Project PIA and verifying the date of approval prior to creating the Project Dataset(s)* | | | | | |
|  | 2020-10-19 | | | | | |
| **Principal Investigator (PI):** | Martin Rotenberg | | | | | |
| **Check the applicable box if the PI is an ICES Student/Trainee** | ICES Student  ICES Fellow  ICES Post-Doctoral Trainee  Visiting Scholar | | | | | |
| **\Responsible ICES Scientist:** | *Name the Responsible ICES Scientist if the PI is not a Full Status ICES Scientist* | | | | | |
|  | Paul Kurdyak | | | | | |
| **Project Team Member(s) Responsible for Project Dataset Creation and/or Statistical Analysis and date joined (list all):** | *All person(s) (ICES Analyst, Appointed Analyst, Analytic Epidemiologist, PI, and/or Student) responsible for creating the Project Dataset(s) and/or statistical analysis on the Research Analytics Environment (RAE) and the date they joined the project must be recorded* | | | | | |
|  | Martin Rotenberg  Paul Kurdyak  Kelly Anderson  Peter Gozdyra | | | yyyy-mon-dd | | |
| **Project Team Member(s) who will request RAE folder access (list all):** | *List the project team member responsible for dataset creation who will request access for all members requiring RAE project folder access (e.g. analyst, methodologist, student).* | | | | | |
|  | Martin Rotenberg | | yyyy-mon-dd | | |  |
| **Other ICES Project Team Members and date joined (list all):** | *All other Research Project Team Members (e.g., Research Administrative Assistants, Research Assistants, Project Managers, Epidemiologists) and the date they joined the project must be recorded* | | | | | |
|  | Martin Rotenberg  Paul Kurdyak  Kelly Anderson  Peter Gozdyra | | | yyyy-mon-dd | | |
| **Confirmation that DCP is consistent with Project Objectives:** | *The following individuals must confirm that the ICES Data provided for in this DCP is relevant (e.g., with respect to cohort, timeframe, and variables) and required to achieve the Project Objectives stated in the ICES Project PIA prior to initial Project Dataset creation: 1) PI; 2) Responsible ICES Scientist if the PI is not a Full Status ICES Scientist, or a second ICES Scientist or the Scientific Program Lead if the PI is creating both the DCP and the Project Dataset[s]; 3) ICES Research and Analysis Staff creating the DCP; and 4) ICES Analytic Staff (ICES Employee or agent responsible for creating the Project Dataset[s]). This may be delegated either verbally or via e-mail.* | | | | | |
|  | ***Principal Investigator*** |  | | | 2020-dec-15 | |
|  | ***Responsible ICES Scientist or Second ICES Scientist/Lead*** |  | | 2020-dec-21 | | |
|  | ***ICES Research and Analysis Staff Creating the DCP*** |  | | yyyy-mon-dd | | |
|  | ***ICES Analytic Staff*** |  | | 2020-dec-16 | | |
| **Designated ICES Research and Analysis Staff accountable for Project Documentation:** | *The person named (ICES staff) is accountable for ensuring that the approved ICES Project PIA, ICES Project PIA Amendments, and DCP are saved on the T Drive, ensuring ICES Project PIA Amendments are submitted as required, ensuring DCP Amendments are documented, and sharing the final DCP with the PI/Responsible ICES Scientist at project completion* | | | | | |
|  |  | | | | | |

| **DCP Creation Date and Author:** | *Date DCP was finalized prior to Project Dataset(s) creation* | *Name of person who created the DCP* |
| --- | --- | --- |
|  | ***Date*** | ***Name*** |
|  | 2020-NOV-05 | Martin Rotenberg |

| ICES Data  This Section must be Completed Prior to Project Dataset(s) Creation | |
| --- | --- |
| *The ICES Employee or agent who is responsible for creating the Project Dataset(s) must ensure that this list includes only data listed in the ICES Project PIA*  *Changes to this list after initial ICES Project PIA approval require an ICES Project PIA Amendment* | *Mandatory for all datasets that are available by individual year* |
| ***General Use Datasets – Health Services*** | ***Years (where applicable)*** |
| OMHRSOMHRS | 2009-2020 |
| CIHI DADCIHI DAD | 2009-2020 |
| OHIP | 2013-2019 |
| NACRS | 2013-2019 |
| ***General Use Datasets – Care Providers*** |  |
| IPDBIPDB | 2013-2019 |
| See listSee list |  |
| ***General Use Datasets – Population*** |  |
| RPDBRPDB | 2009-2019 |
| See listSee list |  |
| ***General Use Datasets – Coding/Geography*** |  |
| PCCFPCCF | 2014-2019 |
| LHINLHIN | 2009 |
| ***General Use Datasets - Facilities*** |  |
| INSTINST | 2014-2019 |
| ***General Use Datasets - Other*** |  |
| ONMARGONMARG | 2016 |
| See listSee list |  |
| ***Controlled Use Datasets*** |  |
| CICCIC | 1985-2019 |
| See listSee list |  |
| ***Other Datasets (including PSD and PDC data)*** |  |
|  |  |

| Project Amendments and Reconciliation | | | |
| --- | --- | --- | --- |
| **ICES Project PIA Amendment History (add additional rows as needed):** | *Privacy approval date* | *Person who submitted amendment* | *Note that any changes to the list of ICES Data or Project Objectives require an ICES Project PIA Amendment* |
|  | ***Date*** | ***Name*** | ***Amendment*** |
|  | yyyy-mon-dd |  |  |
| **DCP Amendment History (add additional rows as needed):** | *Date DCP amended* | *Person who made the DCP amendment* | *Note that any DCP amendments involving changes to the list of ICES Data or Project Objectives require an ICES Project PIA Amendment* |
|  | ***Date*** | ***Name*** | ***Amendment*** |
|  | yyyy-mon-dd |  |  |
| **Date Programs/DCP reconciled** | *The person(s) creating the dataset and/or analyzing the data are responsible for ensuring that the final DCP reflects the final program(s) when the project is completed* | | |
|  | yyyy-mon-dd | | |

| Project Cohort | | |
| --- | --- | --- |
| **Study Design** | Cohort study  Matched cohort study  Case-control study  Cross-sectional study  Other (specify): | |
| **Index Event / Inclusion Criteria**  *(please ensure index event / inclusion criteria are specified with data sources, variables, study period and values or codes)* | **Inclusion criteria:**   - Valid IKN - Resident of Ontario - Valid OHIP card for 5 years prior to cohort inception with no gaps in elgibility - Age 16– 40 - Discharge dates: April 1, 2014 - March 31, 2019 - Discharge diagnosis of Schizophrenia, Schizoaffective Disorder and Psychotic Disorder NOS (non-specific algorithm) based on DSM-IV / ICD-9 codes (295,298) and ICD-10-CA codes (F20, F25, F29)   **Index event for post-discharge outcomes:**  Date of discharge  **Notes:**  Use episodes of care and consider transfers from DAD to OMHRS, OMHRS to DAD, and between OMHRS reporting facilities as one episode.  For readmissions that occur within 7 days consider that discharge the new index and if there is a another readmission after that second readmission then exclude them | |
| **Estimated Size of Cohort** | 7,000 (if sample size is small consider extending to earlier discharge date of 2012 or 2009) | |
| **Exclusions** *(in order)  (common exclusions are listed in grey italics for consideration)* | Step | Description |
|  | 1 | Invalid IKN |
|  | 2 | Invalid birth date(e.g. missing or after index date), Invalid death date (e.g. before index date), Invalid gender (from RPDB) etc. |
|  | 3 | Non-ontario residents at index (substr(prcddablk,1,2) ne ‘35’) |
|  | 4 | Data errors – e.g., death date before admission/discharge |
|  | 5 | Hospitalization LOS LE 3 days |
|  | 6 | Hospitalization LOS >90 days |
|  | 7 | Based on non-specific schizophrenia algorithm, > or = 3 outpatient OHIP billings for codes 295, 298 (from 1991 to hospitalization) or NACRS encounters for the same *(to confirm number of ED encounters needed to excluded)* |
|  | 8 |  |

| Project Time Frame Definitions | | |
| --- | --- | --- |
| Look-back Window  Observation Window  (in which to look for outcomes)  **Index Event Date**  Accrual Window  Max Follow-up Date | |  |
| **Accrual Start/End Dates** | OMHRS and DAD discharge dates: April 1, 2014 – March 31, 2019 |  |
| **Max Follow-up Date** | March 31, 2020 |  |
| **When does observation window terminate?** | 365 days following discharge date from hospitalization  When outcome of interest (rehospitalization), death or end of follow-up period occurs |  |
| **Lookback Window(s)**  *(please ensure lookback windows are defined with start and end dates and in relation to the index event date)* | - 5 years from hospitalization admission date to ensure no previous hospitalizations with discharge diagnosis of inclusion criteria  - 365 days from hospitalization admission date to account for outpatient physician, ER and previous hospitalization prior to hospitalization of interest |  |

| Variable Definitions (add additional rows as needed)  *A few key points to keep in mind:*  *Please ensure codes, data sources, diagnosis types and lookback periods (if applicable) are provided for all definitions listed below and that codes are provided in Excel format. If borrowing codes from another project, please list all the codes here*  *There are maximum number of digits that can be specified using ICES data (ICD 9 CA codes are up to maximum of 4 digits, ICD 10 CA codes are 6 digits, OHIP diagnosis codes are 3 digits)* | | |
| --- | --- | --- |
| **Main Exposure or Risk Factor** | **Distance from place of residence to discharging institution.**  This distance will be ascertained via the following steps:  The location of place of residence will be identified by the RPDB at time of discharge based on PSTLCODE variable and converted to latitude and longitude by the PCCF  The location of the discharging hospital will be identified by either:  i) For OMHRS hospitalizations -  INST variable from OMHRS to link INST # to INST dataset to obtain latitude and longitude from INST dataset variables LAT and LONG.  or  ii) For DAD hospitalizations -  PSTLCODE variable from DAD and converted to latitude and longitude by the PCCF.  *Exclude: GeoDim link flag = 9 *no match to PCCF*  Distance between place of residence and discharging hospital (in Km) will be calculated using the ***%geodistance*** macro from coordinates obtained above and coded as lat_from, long_from, lat_to, long_to.  ***Note****: Alternative measures of distance will be calculated based on i) driving distance and ii) drive time via network analysis in ArcGIS using location of roads and their posted speed limits obtained from the DMTI CanMap spatial road network file. These will be used as part of planned sensitivity analyses.* |  |
| **Primary Outcome Definition** | **A) 4 separate categories categories identified by:**  Within **7 days of discharge** date of hospitalization any outpatient physician visit based on MHAP MHA standard definitions  1. Any outpatient (Location: O, L, H) OHIP visit/consult to a **psychiatrist** [SPEC = 19]  2. Any outpatient (Location: O, L, H) OHIP visit/consult to a **FP/GP** [SPEC = 00]  3. Any outpatient (Location: O, L, H) OHIP visit/consult to a **psychiatrist** [SPEC = 19] or **FP/GP** [SPEC = 00]  4. NO outpatient OHIP visit/consult meeting criteria of 3 categories above  ***Note****: visit = 1 claim/IKN/physnum/servdate (based on 1 claim associated with a single IKN and single PHYSNUM in 1 day)*  ***Exclusion****: To avoid double-counting, exclude all OHIP fee-codes starting with ‘G’ (these are lab codes; e.g., G010 D./T.PROC-LAB.MED.-URINALYSIS) as follows: [substr(FEECODE,1,1) ne 'G']. Include all other fee-codes that occur with a MHA dxcode.*    **B) 4 separate categories categories identified by:**  Within **30 days of discharge** date of hospitalization any outpatient physician visit based on MHAP MHA standard definitions  1. Any outpatient (Location: O, L, H) OHIP visit/consult to a **psychiatrist** [SPEC = 19]  2. Any outpatient (Location: O, L, H) OHIP visit/consult to a **FP/GP** [SPEC = 00]  3. Any outpatient (Location: O, L, H) OHIP visit/consult to a **psychiatrist** [SPEC = 19] or **FP/GP** [SPEC = 00]  4. NO outpatient OHIP visit/consult meeting criteria of 3 categories above  ***Note****: visit = 1 claim/IKN/physnum/servdate (based on 1 claim associated with a single IKN and single PHYSNUM in 1 day)*  ***Exclusion****: To avoid double-counting, exclude all OHIP fee-codes starting with ‘G’ (these are lab codes; e.g., G010 D./T.PROC-LAB.MED.-URINALYSIS) as follows: [substr(FEECODE,1,1) ne 'G']. Include all other fee-codes that occur with a MHA dxcode.* |  |
| **Secondary Outcome Definition(s)** | **Exploratory outcomes:**  1) Date of 1^st^ OMHRS admission or DAD admission (for mental health reason as per MHA definitions below) within 180 days of discharge date of hospitalization  2) Date of 1^st^ OMHRS admission or DAD admission (for mental health reason as per MHA definitions below) within 365 days of discharge date of hospitalization.  ***Identified based on inpatient hospitalization identified by standard MHA definitions:***  **From DAD var DX10CODE1 with the below listed ICD-10-CA codes:**  - Include if DX10CODE1 = F06-F99, OR  - DX10CODE2 to DX10CODE10 = X60-X84, Y10-Y19, Y28 AND DX10CODE1 ne F06-F99  - Include visits with suspect diagnoses (suspect = T)  **From OMHRS standalone dataset up to March 31, 2016:**  -If var AXIS1_DSM4CODE_DISCH1 complete (i.e,. listed diagnosis from below present) use it  - If not, use PROVDX_DSM4CODE_DISCH1  - Exclude OMHRS admissions if:  - AXIS1_DSM4CODE_DISCH1 in: (290.x OR 294.x) **OR**  - AXIS1_DSM4CODE_DISCH1 is missing AND PROVDX_DSM4CODE_ADM1=2  **From OMHRS standalone dataset starting in April 1, 2016:**  - If var DSM5CODE_DISCH1 complete (i.e,. listed diagnosis from below present) use it  If not, use PROVDX_DSM5CODE_ DISCH1  - Exclude OMHRS admissions if:  - DSM5CODE_DISCH1 in: (290.x, 294.x) **OR**  - DSM5CODE_DISCH1 is missing AND PROVDX_DSM5CODE_DISCH1=17  **To identify:**  Dates of all MHA hospitalization admissions (from OMHRS episodes and DAD) in the 365 days after index hospitalization  Dates of all MHA hospitalization discharges  LOS of all MHA hospitalizations, calculated by dates of above |  |
| **Baseline Characteristics** | **Forensic v Non-Forensic hospitalization** – Identify forensic OMHRS admission based on OMHRS variable X9A (values 1 – 9), code as = 1, all others = 0  **OMHRS vs DAD hospitalization** – OMHRS discharges = 1, DAD discharges = 0  **Age** – at discharge from hospitalization, calculated based on date of birth from RPDB and date of discharge from hospitalization, continuous, mean +/- SD  **Sex** – use RPDB variable SEX (1= Male, 2= Female)  **Residential stability** – based on OMHRS_DISCHARGE variable OMHRS_CC5FMT where i) Stable residence ( 0 = Person’s last residence is not considered temporary), and ii) Unstable residence (1 = Person’s last residence is considered temporary)  **Lived alone** – based on OMHRS_DISCHARGE variable OMHRS_CC3FMT where i) Lived alone (1 = Lived Alone), or ii) Did not live alone ( based on 2 = Lived with spouse only 3 = Lived with spouse and other (s), 4 = Lived with child (not spouse), 5 = Lived with other (s) (not spouse or children), 6 = Lived in group setting with non-relative (s))  **Immigrant status** – based on CIC database (CIC_IRCC) where i) General population = not in CIC database, ii) Immigrant = immigrant or iii) Refugee = refugee.  **Country of origin** – categorical variable, from CIC_IRCC database variable WHO_CIC_COUNTRY.  To match categories from: Anderson, K. K., Cheng, J., Susser, E., McKenzie, K. J., & Kurdyak, P. (2015). Incidence of psychotic disorders among first-generation immigrants and refugees in Ontario. Cmaj, 187(9), E279-E286.  **LHIN sub-region** – identified based on RPDB variable at time of discharge  **LHIN region**- identified by RPDB variable LHIN (categorical) at time of discharge  **Material deprivation quintile (DA)** – quintiles based on ONMARG variable DEPRIVATION_Q_DA from DA16.  Based on linkage from a conversion of RPDB variable PSTLCODE via the PCCF to DA11UID.  **Dependency quintile (DA)** – quintiles based on ONMARG variable DEPENDENCY_Q_DA from DA16.  Based on linkage from a conversion of RPDB variable PSTLCODE via the PCCF to DA11UID.  **Ethnic concentration quintile (DA)** – quintiles based on ONMARG variable ETHNICCON_Q_DA from DA16.  Based on linkage from a conversion of RPDB variable PSTLCODE via the PCCF to DA11UID.  **Instability quintile (DA)** – quintiles based on ONMARG variable INSTABILITY_Q_DA from DA16.  Based on linkage from a conversion of RPDB variable PSTLCODE via the PCCF to DA11UID.  **Note:** Due to higher likelihood of data quality issues at the census dissemination area (DA) level also identify in a similar manner as above (ONMARG quintiles) for i) ONMARG Member: LHINSR16 with the additional variable POP_LHINSR, ii) CT16, and iii) CSD18.  **Income Quintile** (neighbourhood) – based on RPDB variable INCQUINT at time of hospitalization discharge  **Rural Residence** – based on RPDP variable RIO2008 where 1 = Rural and 0 = Non-rural  **Diagnosis** – dichotomous, i) Schizophrenia & Schizoaffective Disorder (DSM-IV/ICD-9: 295, or ICD-10-CA: F20, F25) or ii) Psychosis NOS (DSM-IV/ICD-9 : 298, or ICD-10-CA: F29)  Obtained from DAD - Variable: DX10CODE or OMHRS OMHRS - Variable: DSM5CODE_DISCH1 or AXIS1_DSM4CODE_DISCH1  **Substance use** – from OMHRS_ADMISSION variable SUBUSE_MHC where i) Current problems with substance use, and ii) no current problems with substance use    *Note*: this is a summary measure based on OMHRS variables C4a-d, CC2d, C5, C2a-f, and K4. This is not available on the public data dictionary but is available on the intranet.  **Length of stay** – continuous (range 3-90), number of days on either:  i) OMHRS – calculate LOS on discharge date and admission date (based on entire episode of care)  or  ii) DAD – calculate LOS based on discharge date and admission date (entire episode of care)  **Positive Symptoms Scale** – continuous, (range 0-24) from OMHRS variable [CIHI_PSS_LONG](javascript:__doPostBack('ctl00$MainContent$gvList$ctl109$VariableLink','')), where higher scores indicate higher levels of positive symptoms.  **Involuntary admission** – based on OMHRS_ADMISSION variable OMHRS_A3A  Voluntary ( OMHRS_A3A = 1)  Involuntary admission OMHRS_A3A = 4 )  **Insight** – based on OMHRS_DISCHARGE variable B2 where:  0 = Full insight (i.e., the person recognizes that a problem exists and appears to understand the problem or that he or she needs treatment) 1 = Limited insight (i.e., the person acknowledges the problem but may not be able to identify causative factors) 2 = No insight (i.e., the person appears to have no awareness of difficulties or the presence of a mental health problem)  **Any ODB claim in year prior to hospitalization (ODB eligibility)** – if any recorded ODB claim ODB - Variable: SERVDATE, then ODB = 1.  *Note: this will identify those who are potentially eligible to receive funding for publicly funded AP via ODSP, OW*  **Any ODB antipsychotic claim in year prior to hospitalization -** if any recorded ODB claim for the any antipsychotic (AP) medications in the year prior to hospitalization admission date based on DINs in attached spreadsheet.  **Any ODB antipsychotic claim in 30 days following admission** –  As per DINs in spreadsheet.  **Any ODB antipsychotic claim in 180 days following admission** –  As per DINs above  **Any ODB antipsychotic claim in 365 days following admission** –  As per DINs above  **Long acting injectable** – updated Feb 10^th^ 2021 Dichotomus NO/YES, response of YES is based on >= 2 claim of a medication in LONG_ACTING category flagged with “Y” in excel spreadsheet.  Create a flag for each medication time period (year prior to admission, 180 days and 365 days after admission)  For 30 days after admission flag use 1 or more long acting injectable claims to determine use flag  **Teaching hospital** – NO/YES, based on matching of OMHRS variable INST to library INST, dataset TEACHINGINST, variable INST.  **Psychiatrist visit in year prior to admission** – NO/YES, coded YES if any outpatient (Location: O, L, H) OHIP visit/consult to a **psychiatrist** [SPEC = 19] in 365 days prior to admission  **Note**: visit = 1 claim/IKN/physnum/servdate (based on 1 claim associated with a single IKN and single PHYSNUM in 1 day)  **Exclusion**: To avoid double-counting, exclude all OHIP fee-codes starting with ‘G’ (these are lab codes; e.g., G010 D./T.PROC-LAB.MED.-URINALYSIS) as follows: [substr(FEECODE,1,1) ne 'G']. Include all other fee-codes that occur with a MHA dxcode.  **Psychiatrist visit 30 days prior to admission** – NO/YES, coded YES if any outpatient (Location: O, L, H) OHIP visit/consult to a **psychiatrist** [SPEC = 19] in the 30 days prior to admission  **Note**: visit = 1 claim/IKN/physnum/servdate (based on 1 claim associated with a single IKN and single PHYSNUM in 1 day)  **Exclusion**: To avoid double-counting, exclude all OHIP fee-codes starting with ‘G’ (these are lab codes; e.g., G010 D./T.PROC-LAB.MED.-URINALYSIS) as follows: [substr(FEECODE,1,1) ne 'G']. Include all other fee-codes that occur with a MHA dxcode.  **GP visit in year prior to admission** – NO/YES, coded YES if ANY  outpatient (Location: O, L, H) OHIP visit/consult to a **FP/GP** [SPEC = 00] << AND a mental health diagnostic code listed below.>> in 365 days prior to admission  **Note**: visit = 1 claim/IKN/physnum/servdate (based on 1 claim associated with a single IKN and single PHYSNUM in 1 day)  **Exclusion**: To avoid double-counting, exclude all OHIP fee-codes starting with ‘G’ (these are lab codes; e.g., G010 D./T.PROC-LAB.MED.-URINALYSIS) as follows: [substr(FEECODE,1,1) ne 'G']. Include all other fee-codes that occur with a MHA dxcode.  **GP visit 30 days prior to admission** – NO/YES, coded YES if ANY  outpatient (Location: O, L, H) OHIP visit/consult to a **FP/GP** [SPEC = 00] << AND a mental health diagnostic code listed below.>> 30 days prior to admission  **Note**: visit = 1 claim/IKN/physnum/servdate (based on 1 claim associated with a single IKN and single PHYSNUM in 1 day)  **Exclusion**: To avoid double-counting, exclude all OHIP fee-codes starting with ‘G’ (these are lab codes; e.g., G010 D./T.PROC-LAB.MED.-URINALYSIS) as follows: [substr(FEECODE,1,1) ne 'G']. Include all other fee-codes that occur with a MHA dxcode.  ***Note:*** *Steele LS, Glazier RH, Lin E, Evans M. Using administrative data to measure ambulatory mental health service provision in primary care. Medical Care. 2004 Oct;42(10):960-965. DOI: 10.1097/00005650-200410000-00004.*  **ED visit for any mental health issue in the year prior to admission -** From NACRS var DX10CODE1 (with the below listed ICD-10-CA codes) within 365 days prior to admission  -Include if DX10CODE1 = F06-F99, OR  -DX10CODE2 to DX10CODE10 = X60-X84, Y10-Y19, Y28 AND DX10CODE1 ne F06-F99  -Include visits with suspect diagnoses (suspect = T)  ***Note:*** *ICD-9-CM codes displayed as _ _ _.x or _ _.x or ICD-10-CA 3-digit codes (e.g., F10) should include all higher-order digits.*  ***ICD-10-CA (DAD/NACRS) Codes***  **Any mental health and addictions**  DX10CODE1= F06-F99 or DX10CODE2-DX10CODE10 = X60-X84, Y10-Y19, Y28 when DX10CODE1 ne F06-F99  **Substance-Related Disorders**  F55, F10 to F19  **Schizophrenia**  F20 (excluding F20.4), F22, F23, F24, F25, F28, F29, F53.1  **Mood disorders**  F30, F31, F32, F33, F34, F38, F39, F53.0  **Anxiety disorders**  F40, F41, F42, F43, F48.8, F48.9; F93.1, F93.2  **Deliberate self-harm***  DX10CODE2-10 = X60-X84, Y10-Y19, Y28 when DX10CODE1 ne F06-F99  **Deliberate self-harm is an external injury, thus analyst must specify DXTYPE = alldx or DXTYPE = 9 when pulling from DAD.*  **ED visit for any mental health issue 30 days prior to hospitalization -** as above but 30 days prior to admission  **Hospitalization for a mental health reason other than a schizophrenia spectrum disorder or psychosis NOS in the year prior to admission** – based on any hospitalization in 365 days prior to index hospitalization (that results in cohort entry) based on OMHRS and DAD data based on DX10CODE1 (DAD) and AXIS1_DSM4CODE_DISCH1 (OMHRS).  ICD-10 = F10–F51, F53, F55, F59, F60–F69, F91–F99 DSM = All hospitalizations EXCEPT 290, 293, 294, 299, 302, 314–319, 607–787 and V codes |  |
| **P97** | **Year of discharge** – continuous, from either OMHRS variable X80 (discharge date) or DAD variable DDATE (discharge date) |  |

| Analysis Plan and Dummy Tables (expand/modify as needed)  *(please ensure the analysis plan is outlined with dummy tables (can be a separate document)*  *and clear specification of exposures / outcomes / covariates for each model)* | | |
| --- | --- | --- |
| **Descriptive Tables (insert or append dummy tables), e.g.:**  *note: all tables will be based on sample of cohort with OMHRS data*  **** See attached for excel file with each dummy table in an individual worksheet **** | | |
| **Table 1.** Sociodemographic characteristics of the cohort of people with an index hospitalizations for schizophrenia, schizoaffective disorder and psychosis NOS ages 16-40 years in Ontario between 2014-2019. | | |
| **Table 2.** Characterization of primary exposure, follow-up, re-hospitalization and previous service uses by LHIN for a cohort of people with an index hospitalizations schizophrenia, schizoaffective disorder and psychosis NOS ages 16-40 years in Ontario between 2014-2019 | | |
| **Table 3**. Factors associated with follow-up by psychiatrist, GP, or either type of physician within 7 days of discharge after first hospitalization for schizophrenia, schizoaffective disorder and psychosis NOS. | | |
| **Table 4.** Factors associated with follow-up by psychiatrist, GP, or either type of physician within 30 days of discharge after first hospitalization for schizophrenia, schizoaffective disorder and psychosis NOS. | | |
| **Statistical Model(s)** | | |
| **Type of model** | Multilevel (mixed) log-binomial regression model  ****note***: random intercept of sub-LHIN (to account for clustering of data) | |
| **Primary independent variable** | Distance (continuous) - based on Euclidean calculation using macro as described above. | |
| **Dependent variable** | Note: each will be fit with individual adjusted models with covariates below, these models will be **restricted to non-Forensic, OMHRS clients only**   - Follow-up within 7 days of discharge with psychiatrist - Follow-up within 7 days of discharge with GP - Follow-up within 7 days of discharge with either (GP or psychiatrist) - Follow-up within 30 days of discharge with psychiatrist - Follow-up within 30 days of discharge with GP - Follow-up within 30 days of discharge with either (GP or psychiatrist) | |
| **Covariates** | - Age - Sex - Residential stability ( vs. unstable residence) - Lives alone (vs. does not live alone) - Immigration status (immigrant, general population, refugee) - Diagnosis (Schizophrenia & Schizoaffective disorder vs. NOS) - Substance use (current problem with substance use vs. no current problem with substance use) - Length of stay - Teaching hospital (vs. non-teaching) - Psychiatric hospital (vs. general hospital) - Positive symptoms scale - Involuntary admission (vs. voluntary) - Insight ( full, limited, or no insight) - Psychiatrist visit (year prior to hospitalization) - GP visit (year prior to hospitalization) - ED visit for any mental health issues (year to hospitalization) - Hospitalization for a mental health reason other than schizophrenia spectrum d/o or psychosis NOS (during year prior) - Year of discharge (continuous) | |
| **Sensitivity Analyses** | Models with alternative way of measuring primary independent variable - with distance calculated via i) drive time and ii) driving distance (to assess if estimates differ to Euclidean distance) calculated with ArchGIS | |
| **Type of model** | Same as above. | |
| **Primary independent variable** | Model 1: Drive time  Model 2: Driving distance | |
| **Dependent variable** | Same as main analysis. | |
| **Covariates** | Same as main analysis. | |
| **Statistical Model(s)** | | |
| Type of model | *For exploratory objective:*  Multilevel Cox proportional hazard models (frailty models) with LHIN sub-region as a random intercept  *Note: see Austin, P. C. (2017). A tutorial on multilevel survival analysis: methods, models and applications. International Statistical Review, 85(2), 185-203.*  Stratified by 1) No physician follow-up within 30 days, 2) No psychiatrist follow-up within 30 days of discharge and 3) No GP follow-up within 30 days of discharge  *Note: each will be fit with individual adjusted models with covariates below, these models will be restricted to non-Forensic, OMHRS clients only* | |
| Primary independent variable | Time to event outcome*: Date of 1^st^ re-hospitalization (based on OMHRS admission assessment or DAD admission date for mental health admission)  *censor if alive and not hospitalized at 180 days *(consider 365 days based on distribution of data)* | |
| **Dependent variable** | Based on how these hospitalizations are geographically distributed either i) LHIN (rather than sub-region) or i) a KM cut-off based on sample distribution.  ***Note****: this is an exploratory aim of this project*. | |
| **Covariates** | - Age - Sex - Residential stability ( vs. unstable residence) - Lives alone (vs. does not live alone) - Immigration status (immigrant, general population, refugee) - Diagnosis (Schizophrenia & Schizoaffective disorder vs. NOS) - Substance use (current problem with substance use vs. no current problem with substance use) - Length of stay - Teaching hospital (vs. non-teaching) - Psychiatric hospital (vs. general hospital) - Positive symptoms scale - Involuntary admission (vs. voluntary) - Insight ( full, limited, or no insight) - ODB – Antipsychotic ** (as part of sensitivity analysis in cohort that would be ODB eligible)* - ODB – Long-acting antipsychotic ** (as part of sensitivity analysis in cohort that would be ODB eligible)* - Psychiatrist visit (year prior to hospitalization) - GP visit (year prior to hospitalization) - ED visit for any mental health issues (year prior to hospitalization) - Hospitalization for a mental health reason other than schizophrenia spectrum d/o or psychosis NOS (during year to hospitalization) - Year of discharge (continuous) | |
|  | |  |

| Quality Assurance Activities | | | |
| --- | --- | --- | --- |
| **RAE Directory of SAS Programs** |  | | |
| **RAE Directory of Final Dataset(s)** | *The* *final analytic dataset for each cohort includes all the data required to create the baseline tables and run all the models. It should include all covariates for all models such as patient risk factors, hospital characteristics, physician characteristics, exposure measures (continuous, categorical) and outcomes. It should include covariates that were considered but didn’t make the final cut. This would permit an analyst to easily re-run the models in the future.* | | |
|  |  | | |
| **RAE README file available:** Yes No | | | |
| **Date results of quality assurance tools for final dataset shared with project team (where applicable):** | | |  |
|  | | **%assign** | yyyy-mon-dd |
|  | | **%evolution** | yyyy-mon-dd |
|  | | **%dinexplore** | yyyy-mon-dd |
|  | | **%track / %exclude** | yyyy-mon-dd |
|  | | **%codebook** | yyyy-mon-dd |
| **Additional comments:** | |  | |
